# Correction: Abd Algaffar et al. Sandalwood Oils of Different Origins Are Active In Vitro against *Madurella mycetomatis*, the Major Fungal Pathogen Responsible for Eumycetoma. *Molecules* 2024, *29*, 1846

**DOI:** 10.3390/molecules30132763

**Published:** 2025-06-27

**Authors:** Shereen O. Abd Algaffar, Stephan Seegers, Prabodh Satyal, William N. Setzer, Thomas J. Schmidt, Sami A. Khalid

**Affiliations:** 1 Faculty of Pharmacy, University of Science and Technology, Omdurman 14411, Sudan; phd_sh086@hotmail.com; 2 University of Münster, Institute of Pharmaceutical Biology and Phytochemistry (IPBP), PharmaCampus-Corrensstrasse 48, D-48149 Münster, Germany; s_seeg03@uni-muenster.de; 3 Essential Oil Science, dōTERRA International, 1248 W 700 S, Pleasant Grove, UT 84062, USA; psatyal@doterra.com; 4 Department of Chemistry, University of Alabama in Huntsville, Huntsville, AL 35899, USA; wsetzer@chemistry.uah.edu

## Error in Figure 1

In the original publication [1], there was a mistake in Figure 1 as published. The structural diagrams shown represented stereoisomeric forms differing from natural (*Z*)-α-santalol and (*Z*)-β-santalol. The correct absolute configurations of these compounds, according to their entries in the CAS registry (115-17-19 and 42495-69-2, respectively) are shown in the corrected Figure 1 below.

**Figure 1:**
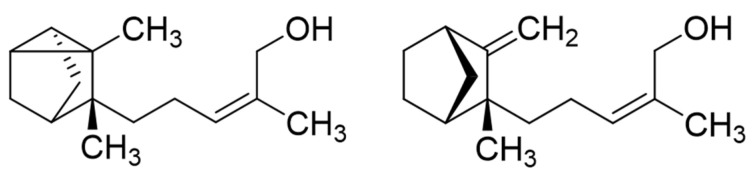
Structures of (*Z*)-α-santalol (left) and (*Z*)-β-santalol (right).

The authors state that the scientific conclusions are unaffected. This correction was approved by the Academic Editor. The original publication has also been updated.
