# Supplementary material for: Sandalwood Oils of Different Origins Are Active In Vitro against Madurella mycetomatis, the Major Fungal Pathogen Responsible for Eumycetoma
Source: Molecules. 2024 Apr 18;29(8):1846. doi: 10.3390/molecules29081846 (PMC11055130; doi:10.3390/molecules29081846)
Supplement: Supplementary file 1 [file molecules-29-01846-s001.zip › molecules-2884096-supplementary.pdf]

molecules

MDPI

Supplementary Materials

Sandalwood oils of different origins are active in vitro against *Madurella mycetomatis*, the major fungal pathogen responsible for eumycetoma

Shereen O. Abd Algaffar[1], Stephan Seegers[2], Prabodh Satyal[3], William N. Setzer[4], Thomas J. Schmidt[2,*] and Sami A. Khalid [1,*]

[1] Faculty of Pharmacy, University of Science and Technology, Omdurman, Sudan; phd_sh086@hotmail.com (S.O.A.A.); khalidseek@hotmail.com (S.A.K.)
[2] University of Münster, Institute of Pharmaceutical Biology and Phytochemistry (IPBP), PharmaCampus – Corrensstrasse 48, D-48149 Münster, Germany; s_seeg03@uni-muenster.de (S.S.), thomschm@uni-muenster.de (T.J.S.)
[3] Essential Oil Science, dōTERRA International, 1248 W 700 S, Pleasant Grove, UT 84062, USA; psatyal@doterra.com (P.S.)
[4] Department of Chemistry, University of Alabama in Huntsville, Huntsville, AL 35899, USA; wsetzer@chemistry.uah.edu (W.N.S.)

* Correspondence: thomschm@uni-muenster.de (T.J.S.); khalidseek@hotmail.com (S.A.K.)

Table S1. Complete list of constituents detected in the Sandalwood oils under study by GC-QTOF MS analysis using a DB-HeavyWax column.

No.	Compound	LRI	EO-24 *S. album*	*Ess-EO1 S. album*	*doT-EO1 S. album 19*	*doT-EO5 S. album (ID)*	*doT-EO6 S. album (ID)*	*doT-EO8 S. album (IN)*	*doT-EO11 S. album (AUS)*	*Ess-EO5 S. paniculatum*	*doT-EO2 S. paniculatum 19*	*doT-EO7 S. paniculatum*	*doT-EO9 S. austrocaledonicum*	*doT-EO10 S. austrocaledonicum*	*Ess-EO4 S. austrocaledonicum*	*Isobionics Biotechn. synthetic*	*Ess-EO2 S. spicatum*	*Ess-EO3 S. lanceolatum*	*Ess-EO6 A. balsamifera*	*Ess-EO7 B. huillensis*
1	α-Copaene	1499																		1,16
2	Ylangene	1506																		1,49
3	n.d.	1522																	0,04	
4	n.d.	1541																		0,15
5	n.d.	1545		0,03	0,04						0,06			0,05						
6	n.d.	1546																	0,03	
7	Sesquithujene	1564								0,03		0,03					0,07	0,11		
8	α-Cedrene	1581																0,45	0,05	
9	α-Santalene	1583	0,94	1,06	0,54	0,47	0,77	0,51	0,86	1,00	1,09	0,70	1,09	1,05	0,71	3,26	0,63			
10	trans-α-Bergamotene	1594	0,15	0,20	0,10	0,10	0,14	0,08	0,14	0,13	0,15	0,09	0,15	0,14	0,09	0,84	0,19			
11	n.d.	1601																	0,03	
12	n.d.	1609																		0,06
13	n.d.	1632																	0,04	
14	n.d.	1639																		0,10
15	epi-β-Santalene	1641	0,99	1,13	0,73	0,58	0,82	0,64	1,02	0,89	0,90	0,64	0,87	0,81	0,55	0,19	0,35			
16	n.d	1643																	0,06	
17	n.d.	1646																		0,12
18	β-Santalene	1655	1,54	1,88	1,13	0,95	1,27	0,99	1,52	1,22	0,91	0,83	0,89	0,81	0,55	1,37	0,52			
19	n.d.	1656																		0,10
20	Muurola-4,5-diene	1657																	0,38	
21	n.d.	1659																0,09		
22	Isocadinene	1665																		0,17
23	α-Acoradiene	1665																	0,90	
24	(E)-β-Farnesene	1670														0,24				
25	n.d.	1676																		0,08
26	Amorpha-4-11-diene	1678																	0,23	
27	β-Acoradiene	1679								0,06		0,04					0,12	0,36		
28	n.d.	1681																		0,05
29	trans-β-Bergamotene	1690														0,16				

30	n.d.	1690																	0,10	
31	α-Amorphene	1694																		10,57
32	γ-Curcumene	1696		0,03						0,10	0,05	0,04	0,06	0,05			0,25	0,53	0,39	
33	γ-Muurolene	1698																		0,24
34	cis-4,10-Epoxyamorphane	1707																		1,05
35	n.d.	1709														0,07				
36	n.d.	1715						0,06				0,03	0,09	0,06						
37	n.d.	1716														0,17				
38	γ-Amorphene	1719																		2,90
39	α-Zingiberene	1724																	0,86	
40	β-Dihydroagarofuran	1726																	0,44	
41	n.d.	1727														0,10				
42	α-Muurolene	1727																		0,79
43	β-Bisabolene	1730	0,06	0,05	0,05	0,06	0,07	0,08	0,05	0,21	0,29	0,08	0,24	0,34	0,24	0,09	0,19	1,85	0,70	
44	γ-Bisabolene	1737	0,02	0,01		0,02	0,02	0,02	0,04								0,03		0,04	
45	β-Curcumene	1743	0,08	0,11	0,07	0,07	0,05	0,07	0,02	0,21	0,12	0,07	0,16	0,13	0,10		0,41	0,78		
46	Sesquicineol	1746															0,11			
47	δ-Cadinene	1759																		3,42
48	7-epi-α-Selinene	1764																	0,25	
49	Tricycloekasantalal	1765	0,08	0,07	0,10	0,09	0,10	0,08	0,17	0,13	0,06	0,10	0,06	0,04	0,04					
50	β-Sesquiphellandren	1771															0,06	0,11	1,18	
51	α-Curcumene	1777	0,35	0,33	0,35	0,24	0,31	0,27	0,43	0,33	0,36	0,31	0,33	0,34	0,26		0,50	0,76	2,05	
52	Selina-3-7-diene	1780																	0,98	
53	n.d.	1783																		0,15
54	n.d.	1793																		0,07
55	n.d.	1808																0,07		
56	n.d.	1818																		0,13
57	cis-Calamenene	1832																		3,83
58	n.d.	1842																		0,72
59	n.d.	1847							0,08	0,02								0,08		
60	n.d.	1853																		0,23
61	n.d.	1857																	0,05	
62	10,11-Epoxycalamenene	1883																		0,28
63	α-Agarofuran	1885																	0,49	

64	γ-Dehydro-ar-Himachalene	1900																		0,65
65	n.d.	1900															0,17	0,06		
66	n.d.	1909				0,02						0,03						0,54		
67	Spirojatamol	1911																		9,25
68	α-Calacorene	1912																		4,43
69	n.d.	1925																		0,55
70	Dendrolasin	1939											0,03	0,05	0,06		1,60	0,68		
71	n.d.	1948																0,43		
72	n.d.	1951	0,03		0,03				0,05											
73	β-Calacorene	1953																		0,28
74	n.d.	1954																0,16		
75	n.d.	1957														0,06				
76	n.d.	1964				0,03				0,05		0,03					0,17	0,32		
77	Caryophyllene-oxide	1965																	0,30	
78	n.d.	1968																	0,33	
79	n.d.	1979																		0,14
80	n.d.	1980				0,02				0,06		0,05					0,06	0,10		
81	n.d.	1986																		0,11
82	n.d.	1991	0,03	0,07			0,06													
83	n.d.	1992																	0,68	
84	n.d.	1997																		0,29
85	α-Photosantalol	2008	0,31	0,38	0,09	0,21	0,35	0,13	0,10	0,25	0,05	0,10	0,04	0,03						
86	n.d.	2008																		0,15
87	n.d.	2011																		0,27
88	n.d.	2012															0,12	0,17		
89	n.d.	2018																	0,13	
90	Gleenol	2032																		0,67
91	n.d.	2034								0,27	0,04	0,18	0,05	0,03						
92	(E)-Nerolidol	2044	0,02	0,02	0,03	0,02	0,04	0,03	0,05	0,10	0,13	0,11	0,14	0,13	0,16		2,72	1,00	0,70	
93	n.d.	2044																		0,09
94	Cubenol	2050																		0,55
95	n.d.	2050																	0,13	
96	α-Corocalene	2054																		0,62
97	Tricycloekasantalol	2055	0,13	0,08	0,15	0,12	0,17	0,07	0,25	0,06	0,08	0,09	0,08	0,07	0,04					
98	Epicubenol	2056																		1,96
99	β-Oplopenone	2059																		2,90
100	n.d.	2062														0,06				

101	Acetyldihydroalbene	2062						0,41			0,20	0,23	0,13	0,15	0,12					
102	5-(2,3-Dimethyltri-cyclo-heptan-3-yl)pentan-2-one	2063	0,37	0,39	0,22	0,24	0,39		0,26	0,22										
103	n.d	2066																	0,21	
104	n.d	2068																		0,16
105	n.d.	2069														0,08				
106	n.d	2074																		0,38
107	α-Elemol	2076																	9,02	
108	n.d.	2080	0,18	0,22	0,06	0,11	0,20	0,10	0,06	0,12	0,04	0,06								
109	Guaiol	2083															0,36			
110	n.d.	2092																		0,30
111	n.d.	2094																		0,16
112	10-epi-γ-Eudesmol	2094																	6,67	
113	n.d.	2098																	0,38	
114	n.d.	2102			0,04					0,05		0,07	0,09	0,06	0,06		0,10	0,08		
115	n.d.	2105																	0,09	
116	Zingiberenol	2109																	0,09	
117	n.d.	2119																		0,15
118	n.d.	2119																0,12		
119	n.d.	2123		0,24												0,08				
120	n.d.	2124																	2,01	
121	Cyclosantalal	2126	0,20	0,18	0,65	0,37	0,28	1,11	0,33	1,37	1,59	1,31	1,65	1,06	0,49					
122	α-Acorenol	2126															0,50	0,27		
123	n.d.	2129																	0,05	
124	n.d.	2134																		
125	epi-Cyclosantalal	2134	0,06	0,10	0,16	0,08	0,07	0,33	0,05	0,27	0,32	0,22	0,32	0,19	0,14					
126	n.d.	2136																		2,20
127	n.d.	2136																	0,40	
128	n.d.	2139																	0,59	
129	n.d.	2141																		0,12
130	Ylangenal	2146																		7,98
131	β-Bisabolol	2148	0,41	0,40	0,51	0,46	0,42	0,40	0,42	0,74	0,55	0,73	0,53	0,50	0,48		2,10	1,99		
132	n.d.	2149																	0,07	
133	n.d.	2154																		0,88
134	n.d.	2154																	0,22	
135	(E)-α-Santalal	2156	1,95	1,44	2,37	1,53	1,44	2,94	2,53	3,03	1,72	2,99	1,40	1,18	0,85	0,21	0,43			
136	γ-Eudesmol	2160																	10,58	
137	n.d.	2161														0,93				

138	n.d.	2162																		0,42
139	n.d.	2164	0,12	0,32	0,15	0,23	0,29	0,24	0,16	0,28	0,11	0,44	0,19	0,16	0,09		0,26	0,22		
140	Copaborneol	2168																		2,81
141	n.d.	2168																	0,53	
142	n.d.	2169		0,09		0,10											0,19			
143	n.d.	2173	0,12		0,62	0,22	0,14	0,45	1,11		0,36	0,30	0,25	0,39	0,33		0,20			
144	n.d.	2174	0,27	0,41		0,34	0,31	0,12		0,41		0,10								
145	n.d.	2176																		
146	τ-Cadinol	2177																	0,52	0,70
147	n.d.	2186	0,07	0,04	0,06	0,06	0,05	0,08	0,14	0,16	0,13	0,25	0,09	0,09	0,08		0,26	0,49		
148	α-Muurolol	2188																		2,13
149	n.d.	2190														0,12				
150	n.d.	2190																	0,68	
151	n.d.	2194																	0,15	
152	n.d.	2196	0,07	0,04	0,18	0,03		0,10	0,28	0,05	0,20	0,17	0,12	0,15	0,09					
153	n.d.	2200																		0,98
154	Bulnesol	2201															0,64			
155	Valerianol	2203																	21,38	
156	Cadalene	2207																		3,85
157	7-epi-alpha-Eudesmol	2208																	6,86	
158	α-Bisabolol	2212	0,06	0,06	0,10	0,20	0,12	0,14	0,10	0,28	0,31	0,19	0,38	0,53	0,58		7,45	2,74		
159	n.d.	2214																	7,18	
160	β-Eudesmol	2215																	11,49	
161	n.d.	2216																		0,33
162	8,14-Cedranediol	2219															0,18	0,42		
163	α-Cadinol	2220																		1,05
164	n.d.	2222	0,17	0,14	0,20	0,15	0,13	0,19	0,25	0,27	0,13	0,36	0,05	0,26	0,03	0,09				
165	n.d.	2228																		0,32
166	n.d.	2232																	0,18	
167	n.d.	2235																0,17		
168	(Z)-β-Santalal	2238	0,55	0,45	0,53	0,38	0,52	0,44	0,81	0,26	0,12	0,22	0,08	0,10	0,10	0,13	0,15			
169	n.d.	2238																	0,20	
170	n.d.	2242																		0,23
171	n.d.	2246														0,90				
172	n.d.	2248																		0,08
173	(E)-β-Santalal	2249	0,12	0,05	0,28	0,10	0,09	0,22	0,48	0,19	0,16	0,23	0,11	0,13	0,10		0,10			
174	n.d.	2249																	0,18	
175	α-Teresantalicacid	2255	0,48	0,50	0,60	0,45	0,49	0,47	0,52	0,57	0,42	0,40	0,42	0,46	0,43		0,43			

176	n.d.	2255																		0,07
177	n.d.	2259																		0,40
178	n.d.	2264																	0,08	
179	n.d.	2268																		0,43
180	n.d.	2271																		0,10
181	Campherenol	2273	0,49	0,54	0,73	0,37	0,55	0,53	0,43	0,49	0,32	0,49	0,30	0,31	0,27		0,44			
182	Bisabolone**	2274																	0,60	
183	n.d.	2282																	0,11	
184	Ylangenol	2287																		11,56
185	Bisabolone**	2288																	0,68	
186	n.d.	2290																		1,41
187	n.d.	2297																		0,26
188	n.d.	2297			0,07	0,07		0,09	0,11	0,29	0,18	0,27	0,19	0,15	0,07		0,46	1,00		
189	n.d.	2298														0,06				
190	n.d.	2303																		0,31
191	n.d.	2303				0,14														
192	n.d.	2305																	0,17	
193	n.d.	2311		0,02		0,02				0,06	0,10	0,05	0,04	0,19	0,21		0,18	0,83		
194	n.d.	2314																	0,40	
195	n.d.	2316																		0,20
196	n.d.	2323																		0,11
197	n.d.	2326	0,03	0,02	0,18	0,15	0,06	0,21	0,17	0,35	0,43	0,47	0,37	0,27	0,15		0,71	0,99		
198	n.d.	2329															0,33	0,75		
199	Humulane-1,6-dien-3-ol	2330																	0,76	
200	(Z)-α-Santalol	2339	49,39	48,23	49,27	46,33	48,46	50,65	48,24	42,36	47,00	45,87	47,47	46,14	45,36	31,90	21,13	0,39		
201	n.d.	2345				0,06						0,14						0,66		
202	(Z)-trans-α-Bergamotol	2350	5,76	6,75	4,35	7,06	6,74	4,61	4,11	4,49	5,03	3,43	5,37	5,66	5,86	2,32	3,46			
203	(2E,6E)-Farnesol	2351								t	t	t	t	t	0,42		10,15	3,35	0,30	
204	4-Isopropyl-6-methyl-1-tetralone	2353																		1,35
205	n.d.	2355										0,10					0,15	0,57		
206	n.d.	2357																	0,74	
207	n.d.	2363															0,54	1,20		
208	n.d.	2365	0,13	0,08	0,21	0,25	0,10	0,17	0,17	0,33	0,38	0,51	0,19	0,29	0,06		1,85	1,78		
209	n.d.	2365														0,43				
210	n.d.	2366																		0,21
211	8-Cedrene-13-ol	2371				0,10				0,22	0,05	0,22	0,05	0,03	0,04		0,32	0,65		

212	(E)-α-Santalol	2379	0,36	0,36	0,32	0,27	0,29	0,29	0,28	0,16	0,31	0,28	0,28	0,27	0,25	15,69	0,24			
213	(E)-trans-α-Bergamotol	2385	0,14	0,19	0,15	0,27	0,22	0,12	0,08	0,09	0,17	0,13	0,14	0,18	0,10	1,64	0,28	0,28		
214	n.d.	2387																		0,34
215	(Z)-epi-β-Santalol	2406	3,24	3,35	4,45	3,01	3,15	3,98	3,83	3,48	3,51	3,93	3,47	3,30	3,39	5,31	1,70	0,03		
216	(Z)-β-Santalol	2420	22,20	21,98	20,23	21,95	20,75	21,12	20,64	16,10	16,74	15,27	18,17	17,35	18,87	18,87	7,94	0,22		
217	n.d.	2423																		0,28
218	n.d.	2424																	0,49	
219	n.d.	2430	0,75	0,82	0,69	0,51	0,55	1,06	0,59	0,68	0,44	0,57	0,54	0,57	0,71					
220	n.d.	2434																	0,11	
221	(Z)-γ-Curcumen-12-ol	2435				0,11				0,99	0,28	0,28	0,24	0,23	0,22		2,84	7,18		
222	n.d.	2439																		0,14
223	n.d.	2440								0,04							0,26	0,46		
224	n.d.	2444															0,15	0,34		
225	(E)-epi-β-Santalol	2448	0,19	0,19	0,20	0,13	0,16	0,21	0,16	0,08	0,07	0,15	0,07	0,11	0,11	2,14	0,21			
226	n.d.	2457								t	0,54	t	0,62	0,54	0,36		0,99	0,92		
227	(E)-β-Santalol	2460	1,55	1,68	1,14	1,72	1,41	1,22	0,97	0,96	t	1,01	t	t	t	7,35	t			
228	n.d.	2465	0,09	0,23		0,36	0,16	t	t		0,22		0,23	0,23	0,26		t	t		
229	n.d.	2468	0,07	0,15		0,25				0,72		0,65			0,13		0,76	3,20		
230	n.d.	2474																		0,16
231	(Z)-β-Curcumen-12-ol	2482	0,11	0,19	0,11	0,56	0,21	0,12		1,75	0,73	0,76	0,68	0,54	0,50		5,97	6,33		
232	(Z)-Lanceol	2487	1,15	1,13	1,64	2,54	2,85	1,65	1,65	3,50	6,82	2,34	6,09	9,24	11,45	0,58	3,02	30,75		
233	Drim-7-en-11-ol	2487																	3,31	
234	n.d.	2488																		0,36
235	n.d.	2505								0,07		0,06			0,29	0,49		0,43		
236	(Z)-Nuciferol	2517	0,74	1,05	1,50	3,54	2,31	0,87	1,53	5,54	2,67	7,90	2,38	2,14	1,92	0,71	11,34	19,87		
237	n.d.	2524	0,08	0,09	0,20	0,11	0,08	0,21	0,10	0,26	0,55	0,22	0,46	0,44	0,39		0,92	1,09		
238	Spirosantalol	2532	0,80	0,98	0,79	0,72	1,02	0,66	0,76	2,01	0,52	1,70	0,52	0,61	0,63		0,77			
239	n.d.	2532																		0,27
240	n.d.	2547														0,55				
241	n.d.	2554			0,20	0,13	0,17		0,66											
242	(E)-Nuciferol	2559	0,11	0,09	0,24	0,28	0,27	0,08	0,73	0,09	0,18	0,07	0,13	0,15	0,05	0,19	0,26	0,25		
243	n.d.	2569																		0,17
244	14-Hydroxy-δ-Cadinene	2572																		0,30

245	n.d.	2583				0,02						0,04								
246	n.d.	2593														0,25				
247	n.d.	2610		0,16		0,10	0,16	0,09		0,13										
248	n.d.	2613																		0,09
249	n.d.	2621																		0,24
250	8-Ketoylangenal	2630																		1,71
251	8-Ketocopaenal	2657																		1,31
252	n.d.	2667																		0,27
253	α-Teresantalic acid	2675	0,29	0,33		0,22	0,31		0,16	0,09										
254	n.d.	2693														0,07				
255	n.d.	2698																		0,12
256	n.d.	2703																		0,15
257	n.d.	2706																	0,06	
258	n.d.	2709					0,03					0,01							0,03	
259	n.d.	2762																		0,10
260	n.d.	2798																		0,39
261	n.d.	2824																		0,21
262	n.d.	2828														0,18				
263	n.d.	2880																		0,10
264	n.d.	2891											0,11	0,08						
265	n.d.	2911								0,08	0,43		0,15	0,19				0,29		
266	n-Hexadecanoicacid	2938	0,95	0,76	1,09	0,42	0,91	0,81	0,93	0,74	1,24	0,63	0,97	0,80	0,57	1,36	1,05	1,08	0,59	0,96
267	n.d.	2983	0,48																	
268	n.d.	3008		0,17	0,29				0,75				0,32							
269	n.d.	3116			0,73															
270	n-Octadecanoicacid	3152	0,72		1,11	0,21		0,52	0,50	0,50	0,49	0,37	0,38	0,48	0,62	0,77			0,30	
271	n.d.	3163										0,23								
272	n.d.	3190																		
273	n.d.	3197																		
274	n.d.	3214	0,30						0,22											

Table S2. Complete list of constituents detected in the Sandalwood oils under study by GC-QTOF MS analysis using a DB-5 column.

No.	Compound	LRI	*S. album* *EO-24*	*S. album* *Ess-EO1*	*S. album 19* *doT-EO1*	*S. album (ID)* *doT-EO5*	*S. album (ID)* *doT-EO6*	*S. album (IN)* *doT-EO8*	*S. album (AUS)* *doT-EO11*	*S. paniculatum* *Ess-EO5*	*S. paniculatum 19* *doT-EO2*	*S. paniculatum* *doT-EO7*	*S. austrocaledonicum* *doT-EO9*	*S. austrocaledonicum* *doT-EO10*	*S. austrocaledonicum* *Ess-EO4*	*Biotechn. synthetic* *Isobionics*	*S. spicatum* *Ess-EO2*	*S. lanceolatum* *Ess-EO3*	*A. balsamifera* *Ess-EO6*	*B. huillensis* *Ess-EO7*
1	n.d.	1296											0,11	0,15						
2	n.d.	1303											0,11	0,13						
3	n.d.	1307											0,11	0,13						
4	n.d.	1323							0,17				0,23	0,26						
5	Tricycloekasantalal	1346	0,09	0,08	0,15	0,12	0,12		0,19	0,15					0,05					
6	Isoledene	1377																		0,40
7	Ylangene	1380																		1,30
8	α-Copaene	1385																		1,06
9	n.d.	1386								0,03										
10	n.d.	1395		0,03																
11	n.d.	1397								0,05					0,04					
12	n.d.	1400							0,45				0,62	0,66						
13	n.d.	1409															0,08	0,16		
14	n.d.	1410											0,13							
15	cis-α-Bergamotene	1419														0,02				
16	n.d.	1420							0,12											
17	α-Cedrene	1423															0,11	0,47		
18	α-Santalene	1425	1,03	1,31	0,60	0,56	0,72	0,54	0,88	1,21	1,14	0,86	1,09	1,10	0,79	4,56	0,68			
19	trans-α-Bergamotene	1438	0,19	0,23	0,13	0,13	0,15		0,14	0,16		0,12	0,15	0,14	0,11	0,88	0,24			
20	epi-β-Santalene	1451	1,21	1,33	0,77	0,63	0,87	0,69	1,14	1,13	0,99	0,76	0,92	0,85	0,65	0,24	0,43			
21	Muurola-4,5-diene	1455																	0,35	
22	(E)-β-Farnesene	1455														0,06				
23	n.d.	1459							0,18				0,27	0,22						
24	n.d.	1460																	0,07	
25	β-Santalene	1463	1,83	2,02	1,10	1,04	1,25	1,08	1,55	1,47	1,04	0,97	0,92	0,83	0,62	1,51	0,63			
26	α-Acoradiene	1465																	1,05	
27	β-Acoradiene	1470															0,15	0,42		
28	n.d.	1471														0,09				

29	n.d.	1472	0,04	0,04				0,12		0,07			0,21	0,19	0,05					
30	n.d.	1480																	0,09	
31	γ-Muurolene	1481																		0,34
32	γ-Curcumene	1482	0,02							0,14					0,03		0,21	0,56	0,22	
33	Amorpha-4-11-diene	1484																	0,14	
34	n.d.	1485														0,09				
35	α-Amorphene	1485																		12,09
36	α-Curcumene	1486	0,40	0,39	0,37	0,28	0,33	0,29	0,43	0,37	0,39	0,39	0,31	0,35	0,26		0,53	0,84	2,08	
37	cis-4-10-Epoxyamorphane	1487																		0,80
38	trans-β-Bergamotene	1489		0,03												0,09				
39	n.d.	1490																	0,08	
40	n.d.	1491														0,07				
41	n.d.	1496							0,40				0,55	0,64						
42	10,11-Epoxycalamenene	1497																		0,75
43	α-Zingiberene	1499								0,05							0,13	0,69	0,73	
44	Pentadecane	1500												0,22					0,49	
45	γ-Amorphene	1501																		2,76
46	n.d.	1502														0,02				
47	n.d.	1504														0,04				
48	α-Muurolene	1505																		1,25
49	n.d.	1505																0,15		
50	β-Dihydroagarofuran	1508																	0,57	
51	β-Bisabolene	1511	0,04	0,05	t	0,08	0,07	0,03	t	0,12	0,31	0,08	t	t	0,25	0,05	0,11	1,87	0,75	
52	2,4-di-tert-butyphenol	1511			0,07	t	t	t	1,26		t	t	1,83	1,85						
53	β-Cadinene	1512																		0,48
54	β-Curcumene	1514	0,11	0,15	0,06	0,12	0,09	t	t	0,26	0,16	0,11	0,15	0,16	0,10		0,53	1,30		
55	n.d.	1515																	0,52	
56	n.d.	1516														0,02				
57	Sesquicineole	1517															0,23	0,16		
58	γ-Cadinene	1519																		0,72
59	n.d.	1519																0,24		
60	5-(2,3-Dimethyltri-cyclo-heptan-3-yl)pentan-2-one	1521	0,33	0,39		0,23	0,34	0,12		0,29										

61	7-epi-α-Selinene	1522																	0,22	
62	cis-Calamenene	1526																		6,93
63	β-Sesquiphellandren	1526															0,08	0,18	1,18	
64	Cypernepoxide	1535																		0,27
65	n.d.	1537																	0,10	
66	n.d.	1539																	0,10	
67	7-methy-Pentadecane	1539										0,25	0,34							
68	γ-Dehydro-ar-Himachalene	1542																		0,67
69	n.d.	1543	0,38	0,44	0,17	0,28	0,24	0,18		0,25					0,05		0,12			
70	3,7-Selinadiene	1544																	1,02	
71	α-Calacorene	1546																		5,30
72	n.d.	1546																0,09		
73	α-Agarofuran	1549																	0,27	
74	n.d.	1550																0,10		
75	α-Elemol	1551																	8,51	
76	n.d.	1552																		0,83
77	n.d.	1555	0,17	0,22		0,12	0,19													
78	n.d.	1556																	0,11	
79	(E)-Nerolidol	1564	0,17	0,10	0,19	0,16	0,19	0,11	0,27	0,15	0,29	0,22	0,20	0,19	0,19		2,74	0,94	0,60	
80	Dendrolasin	1573								0,06					0,07		1,95	0,87		
81	n.d.	1576																		0,46
82	n.d.	1576															0,12			
83	n.d.	1577																	0,08	
84	n.d.	1580		0,06																
85	n.d.	1582																		0,22
86	Acetyldihydroalbene	1584	0,06		0,15			0,33	0,18	0,05	0,22	0,22	0,13	0,11	0,14					
87	Gleenol	1587																		0,82
88	n.d.	1588														0,38				
89	n.d.	1588								0,08										
90	n.d.	1596	0,06	0,04					0,11						0,01					
91	Spirojatamol	1597																		10,77
92	n.d.	1599																0,14		
93	Guaiol	1600															0,38			
94	Hexadecane	1600							0,18				0,21	0,24						
95	n.d.	1603																0,14		
96	5-epi-7epi-α-Eudesmol	1604																	0,59	

97	Copaborneol	1607																		1,86
98	n.d.	1608																	0,78	
99	β-Oplopenone	1610																		3,62
100	n.d.	1613																		0,30
101	n.d.	1614																	0,15	
102	n.d.	1615								0,18										
103	n.d.	1617														0,02				
104	n.d.	1619																		0,72
105	10-epi-γ-Eudesmol	1620																	6,73	
106	α-Corocalene	1622																		0,82
107	n.d.	1624																	2,80	
108	n.d.	1624															0,26	0,51		
109	n.d.	1627														0,49				
110	Epicubenol	1627																		2,80
111	α-Acorenol	1629															0,28	0,20		
112	n.d.	1630																		1,31
113	γ-Eudesmol	1630																	10,36	
114	n.d.	1631																		3,17
115	Hinesol	1637																	0,30	
116	Cubenol	1639																		0,53
117	τ-Cadinol	1640																	0,98	1,97
118	n.d.	1644																		0,85
119	n.d.	1644															0,09			
120	n.d.	1646																	0,08	
121	Ylangenol	1647																		10,80
122	β-Eudesmol	1648																	8,34	
123	n.d.	1648								0,19										
124	α-Muurolol	1649																		1,92
125	Valerianol	1650																	34,73	
126	n.d.	1651	0,21	0,44	0,15	0,29	0,25	0,31		0,42		0,28	0,15	0,14	0,16					
127	α-Cadinol	1652																		1,42
128	n.d.	1652															0,18			
129	7-epi-α-Eudesmol	1655																	9,75	
130	n.d.	1656	0,05		0,47	0,30	0,27	0,26	0,35		0,23				0,06					
131	Ylangenal	1659																		8,81
132	n.d.	1659																0,13		
133	n.d.	1661		0,32																
134	n.d.	1663		0,18	0,33	0,23	0,44		0,53					0,19			0,13			
135	Bulnesol	1665															0,50			

137	n.d.	1666																		0,30
138	Campherenol	1667	0,06	0,27	0,39	0,44	0,41	0,35	0,34	t	0,32	0,84	0,24	0,19	0,07		t			
139	Cyclosantalal	1668	0,39	0,30	0,59	0,31	0,28	1,32	0,50	1,14	1,86	1,52	1,58	1,30	1,30					
140	β-Bisabolol	1669	t	t	t	t	t	t	t	t	t	t	t	t	t		3,06	3,35		
141	(Z)-α-Santalol	1673	58,70	57,30	54,53	51,99	52,19	57,25	53,05	52,69	51,64	53,42	51,60	48,50	53,40	33,33	20,80	1,10		
142	Cadalene	1673																		4,45
143	n.d.	1674																	0,38	
144	n.d.	1676	0,04	0,50						0,11				0,14						
145	n.d.	1681																	0,71	
146	n.d.	1683																		0,32
147	α-Bisabolol	1685	0,07	0,07	t	0,09	0,10	t	0,16	t	t	t	t	0,47	0,56		9,60	3,04		
148	epi-Cyclosantalal	1686	t	t	0,48	t	t	0,97	t	0,83	1,20	1,26	1,00	0,96	0,29					
149	n.d.	1687																	0,14	
150	trans-α-Bergamotol	1690	4,80	5,55	4,08	6,91	6,35	4,60	4,04	4,17	5,58	3,29	5,21	5,49	4,66	1,49	3,78			
151	n.d.	1692																		0,22
152	Selin-7(11)-en-4-ol	1694																	0,10	
153	(E)-α-Santalol	1695	0,30	0,18	0,39	0,20	0,23	0,35	0,17	0,12	0,20	0,24	0,12	0,17	0,03	16,84	0,21			
154	n.d.	1699															0,09	0,16		
155	4-Isopropyl-6-methyl-1-tetralone	1700																		1,34
156	n.d.	1700																	0,59	
157	(Z)-epi-β-Santalol	1702	3,30	2,86	4,62	3,49	3,53	4,32	4,27	3,93	3,92	4,18	3,56	3,32	3,01	5,23	1,35	0,23		
158	n.d.	1708							0,35				0,50	0,56						
159	(E)-trans-α-Bergamotol	1709	0,47	0,19	0,70			0,61	1,08	0,27	0,54		0,42	0,43	0,34	0,76	0,25			
160	n.d.	1711															0,46	1,15		
161	(Z)-β-Santalol	1714	21,25	20,41	23,75	22,70	23,29	22,16	21,76	15,15	17,46	16,19	17,46	16,93	17,86	23,15	7,73	0,45		
162	(2E,6E)-Farnesol	1720								0,29	0,31	t	0,17	0,19	1,19		14,57	3,27	0,15	
163	(E)-epi-β-Santalol	1722														2,49				
164	(Z)-Nuciferol	1723	1,09	1,26	1,94	4,33	2,76	1,22	1,58	7,31	3,86	9,72	2,63	2,49	2,17		16,36	29,91		
165	n.d.	1730																		0,29
166	n.d.	1732																0,13		
167	(E)-β-Santalol	1734	1,54	1,81	1,27	1,13	1,23	0,95	0,92	0,44	0,20	0,58	0,16	0,10	0,08	7,79	0,17			
168	Spirosantalol	1735	0,22	0,29	0,54	1,04	1,02	0,55	0,55	1,49	0,75	2,47	0,59	0,56	0,26		0,52			
169	Bisabolone**	1735																	0,41	

170	n.d.	1738																0,48		
171	Bisabolone**	1741																	0,53	
172	n.d.	1742		0,07			0,22									0,02	0,38			
173	n.d.	1746	0,18		0,54		0,22		0,98	0,33										
174	n.d.	1747																1,46		
175	n.d.	1748								0,38			0,28	0,39	0,12		0,36			
176	n.d.	1750					0,23		0,19				0,22	0,24						
177	(Z)-β-Curcumen-12-ol	1753	0,11	0,18	0,06	0,35	0,12	0,02		1,59	0,58	0,48	0,46	0,29	0,30		5,74	9,90		
178	Drim-7-en-11-ol	1754																	1,92	
179	(Z)-Lanceol	1756	0,99	0,89	0,98	2,46	2,33	1,27	1,32	2,78	6,83	1,81	4,91	7,93	10,55	0,26	2,47	32,86		
180	n.d.	1764																		0,24
181	(E)-Nuciferol	1766															0,29	0,79		
182	n.d.	1773																0,10		
183	n.d.	1775	0,11		0,20															
184	n.d.	1776																	0,10	
185	n.d.	1778																0,35		
186	n.d.	1787							0,17								0,21	0,19		
187	n.d.	1790			0,24					0,08			0,21	0,20	0,21		0,68	0,62		
188	n.d.	1795																0,13		
189	14-Hydroxy-δ-Cadinene	1797																		0,23
190	n.d.	1804																0,17		
191	8-Ketocopaenal	1807																		1,61
192	8-Ketoylangenal	1821																		1,62
193	n.d.	1837		0,05																
194	n.d.	1866																	0,07	
195	n.d.	1879																		0,26

t=tentative

**= stereoisomer not determined

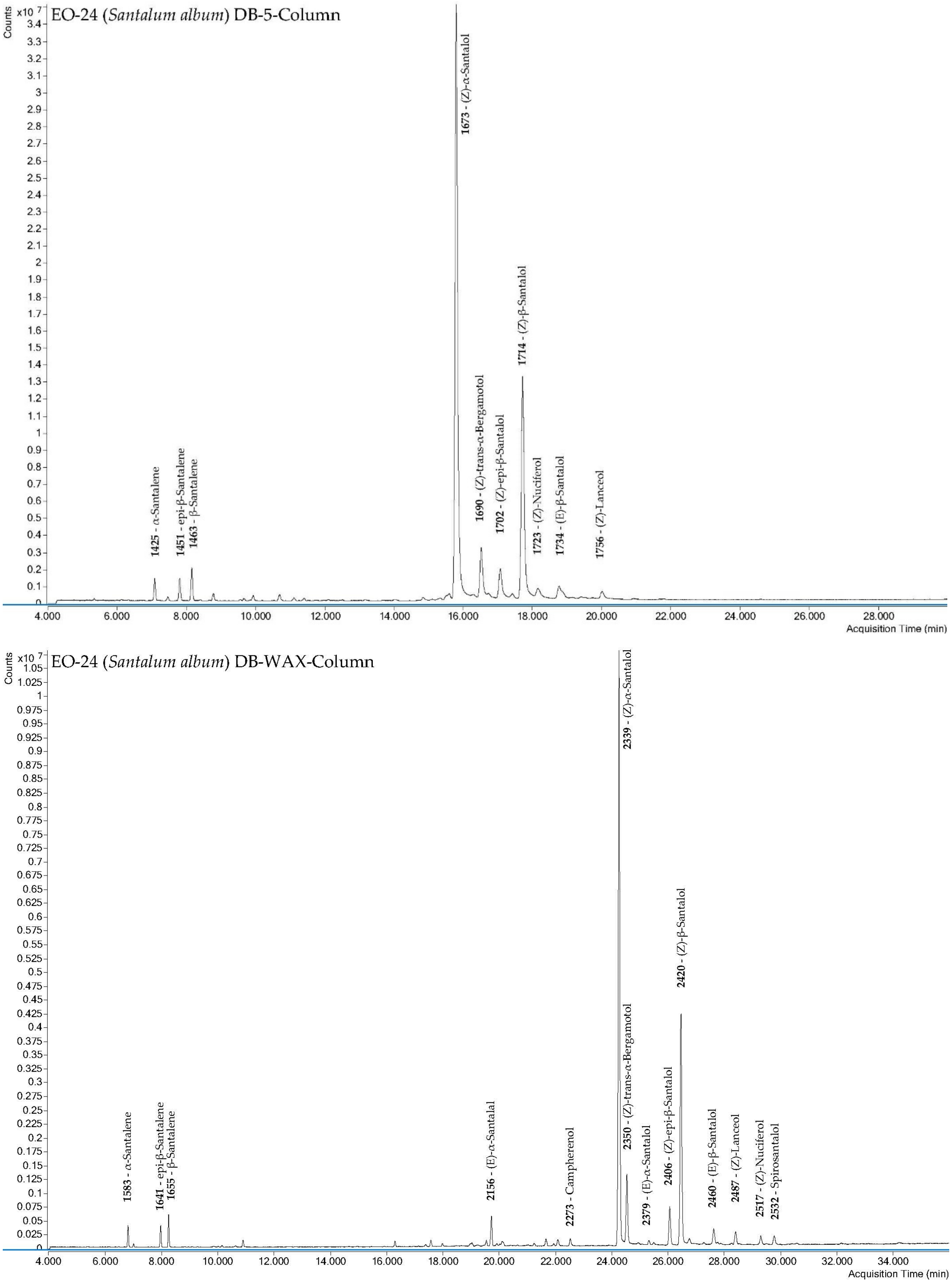

Figure S1. GC-MS chromatograms of EO-24 on DB-5 and DB-HeavyWAX-columns

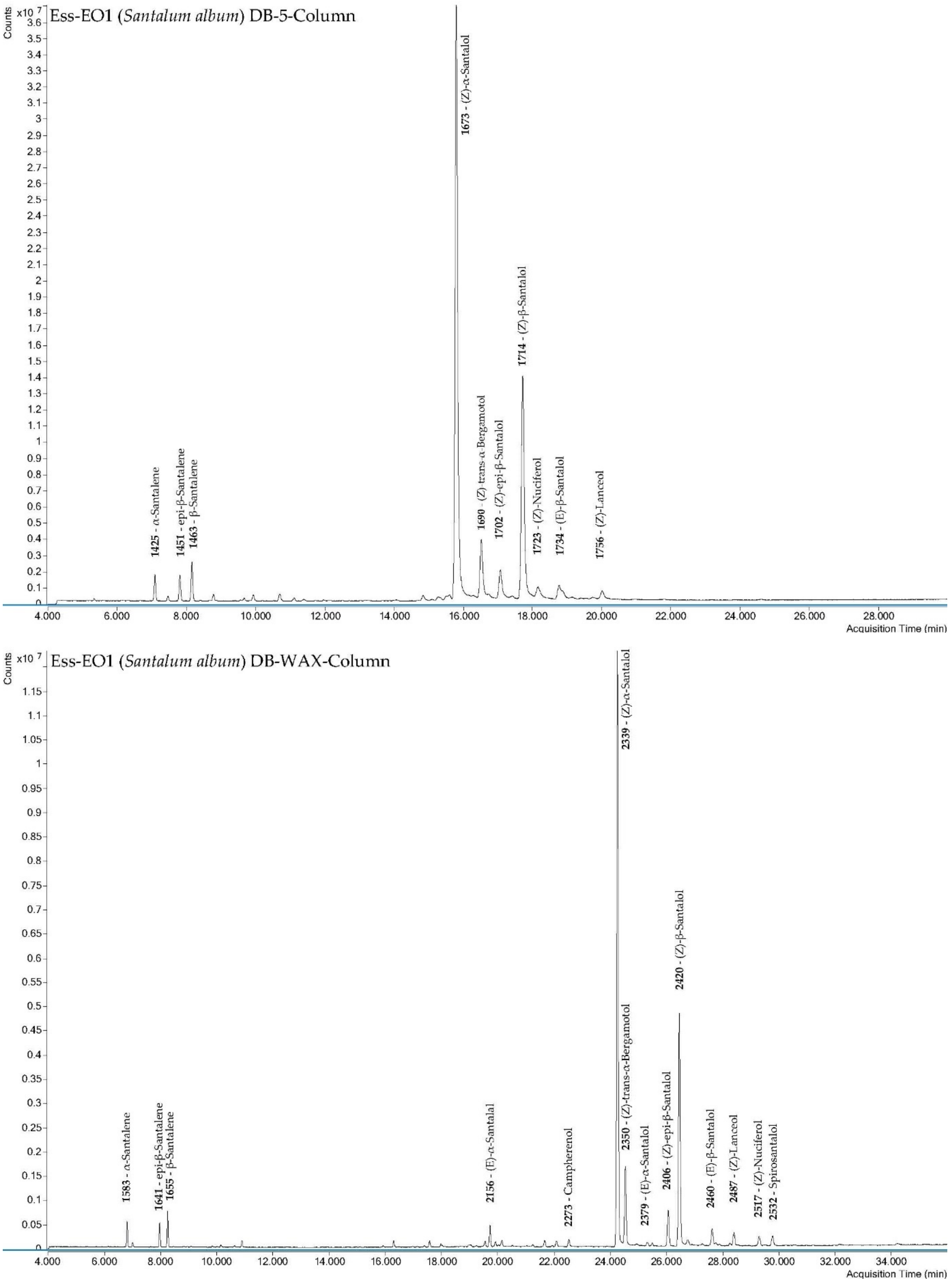

Figure S2. GC-MS chromatograms of Ess-EO1 on DB-5 and DB-HeavyWAX-columns

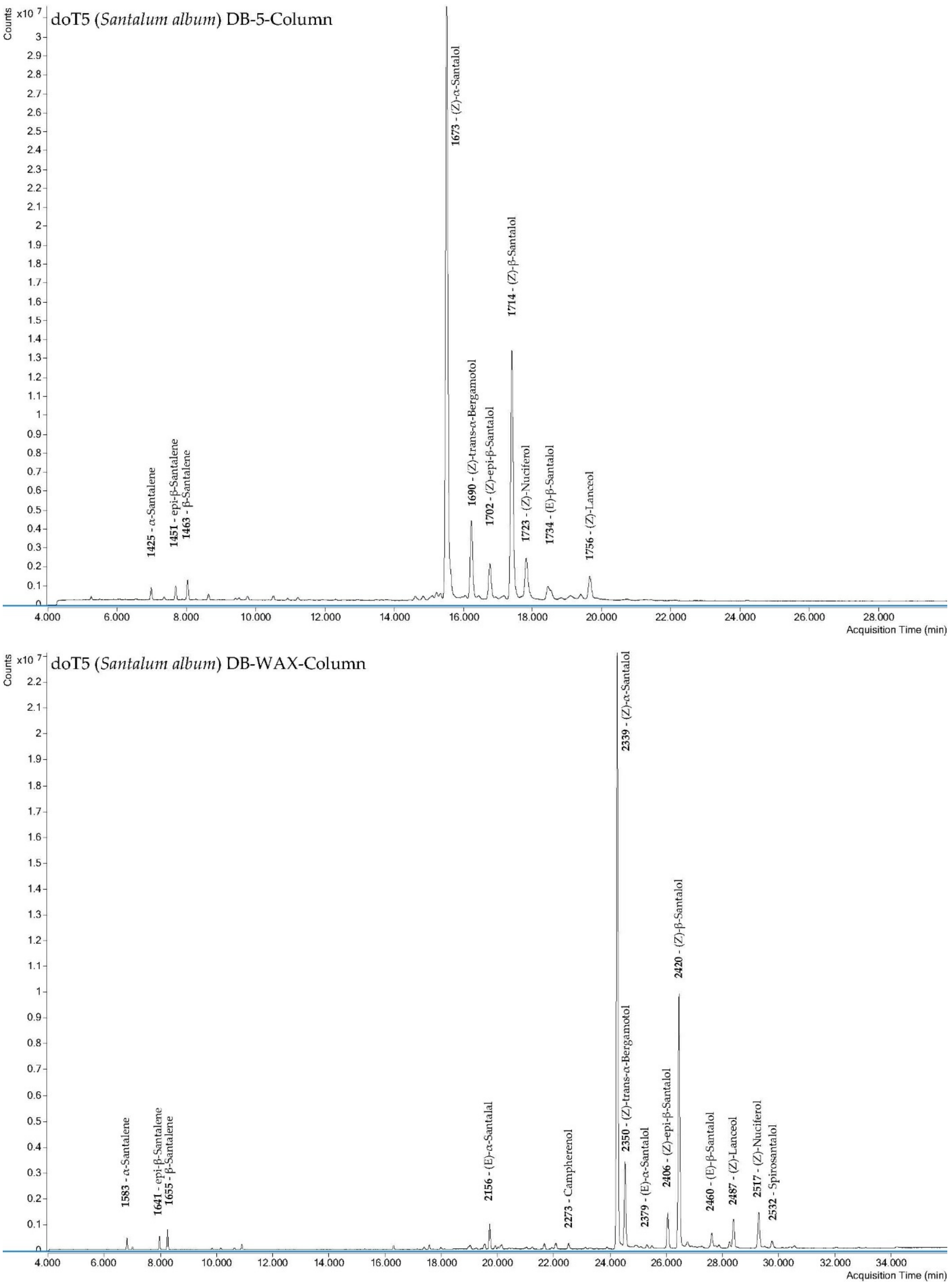

Figure S3. GC-MS chromatograms of doT5 on DB-5 and DB-HeavyWAX-columns

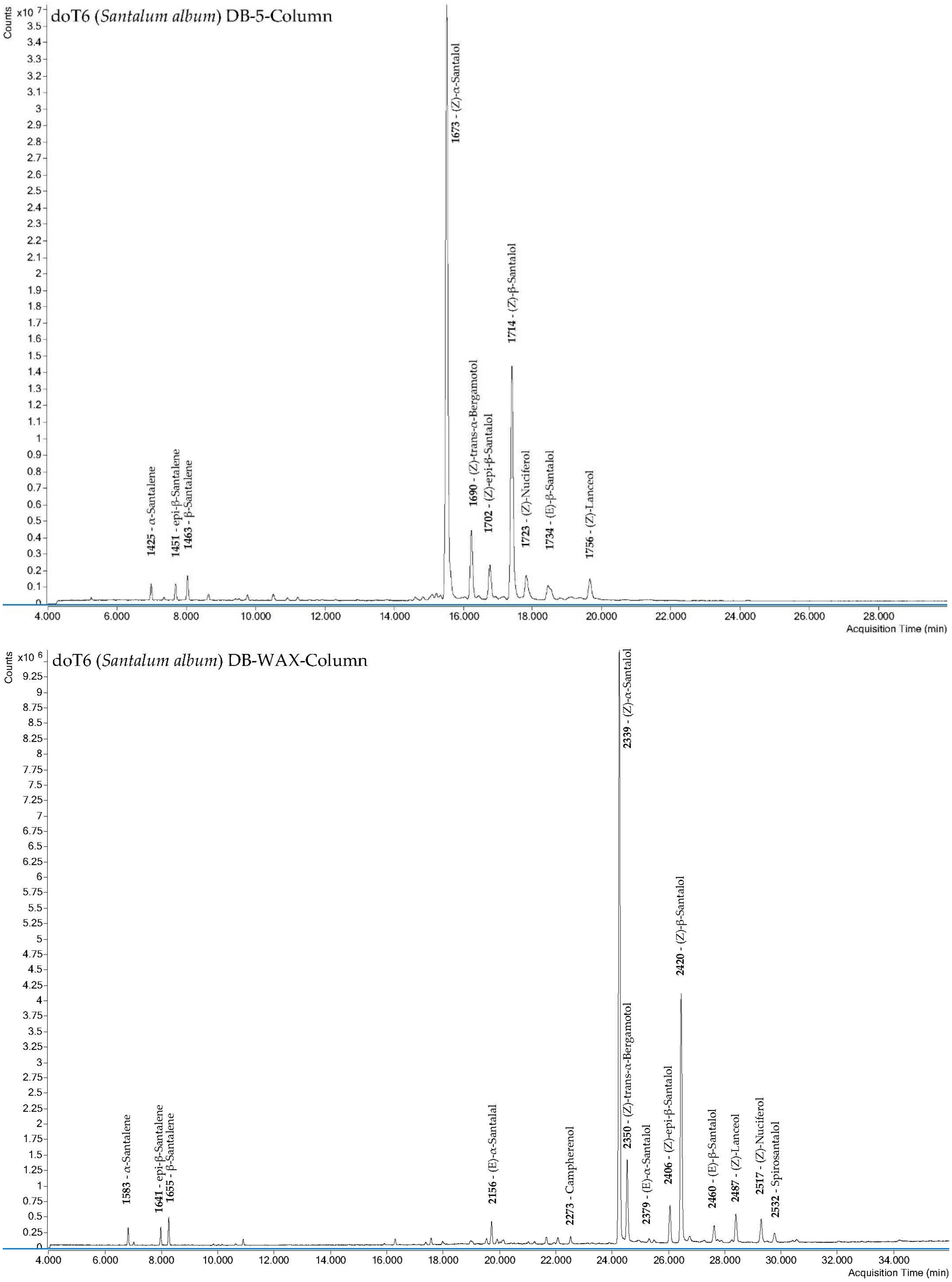

Figure S4. GC-MS chromatograms of doT6 on DB-5 and DB-HeavyWAX-columns

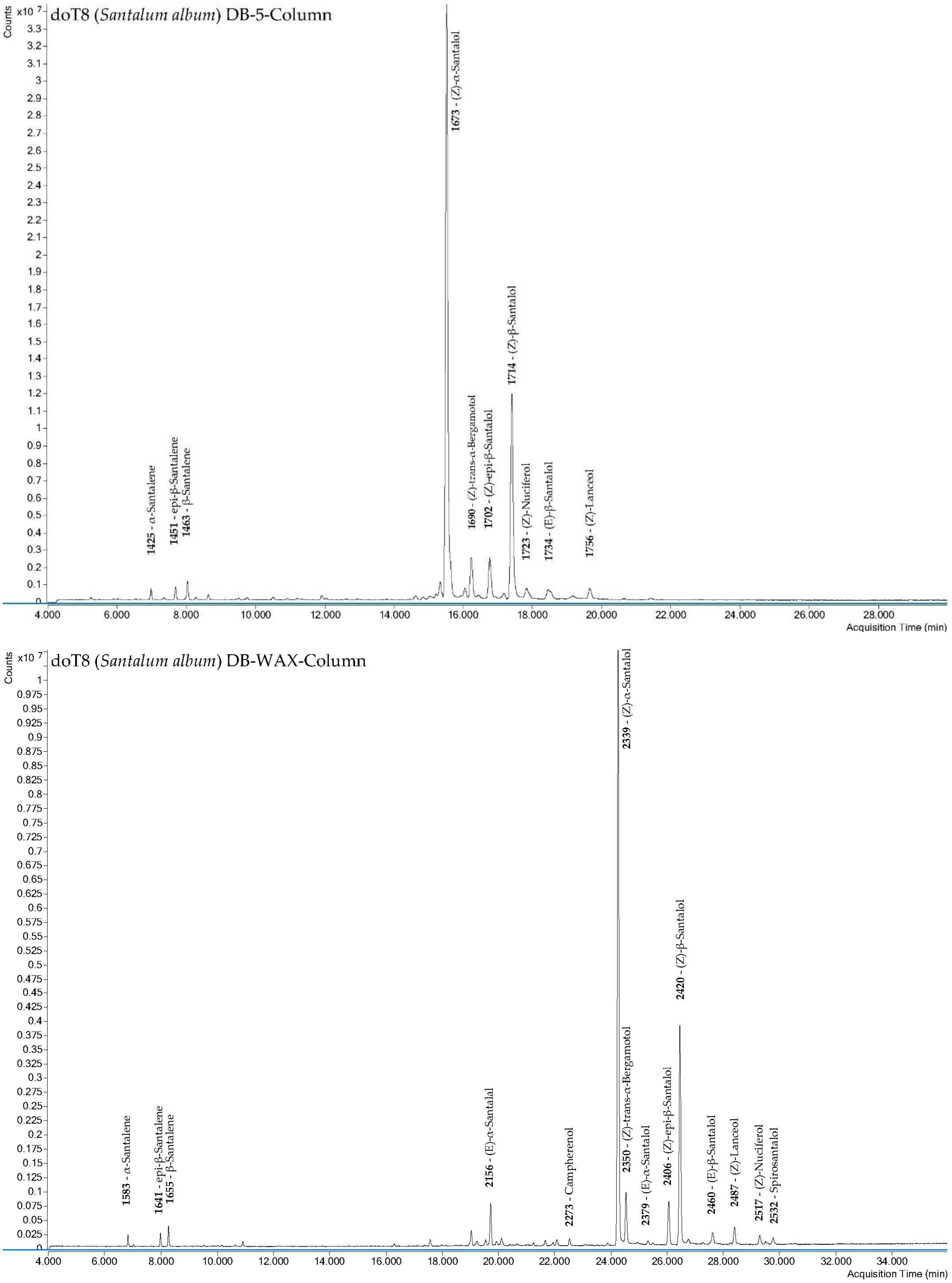

Figure S5. GC-MS chromatograms of doT8 on DB-5 and DB-HeavyWAX-columns

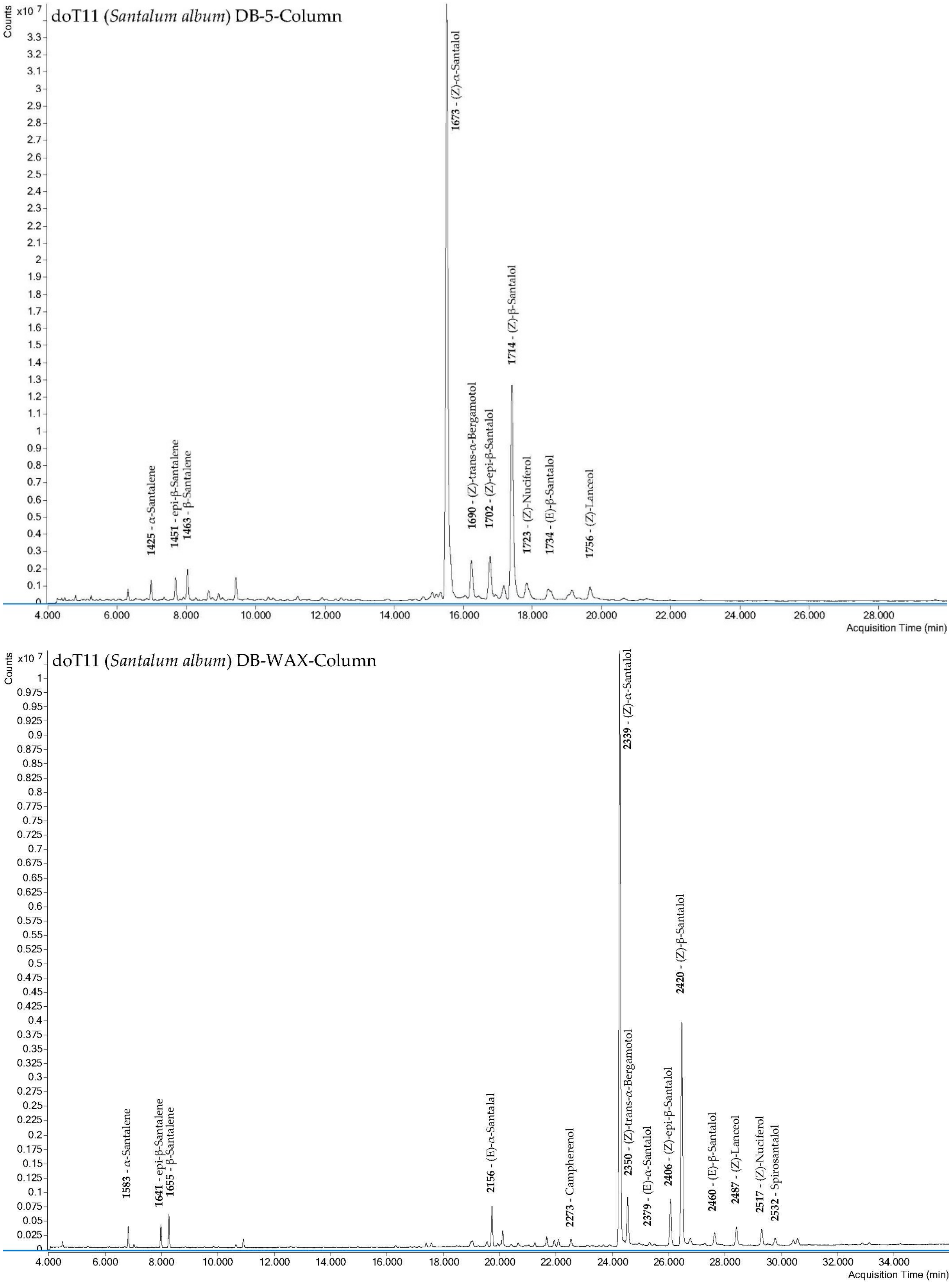

Figure S6. GC-MS chromatograms of doT11 on DB-5 and DB-HeavyWAX-columns

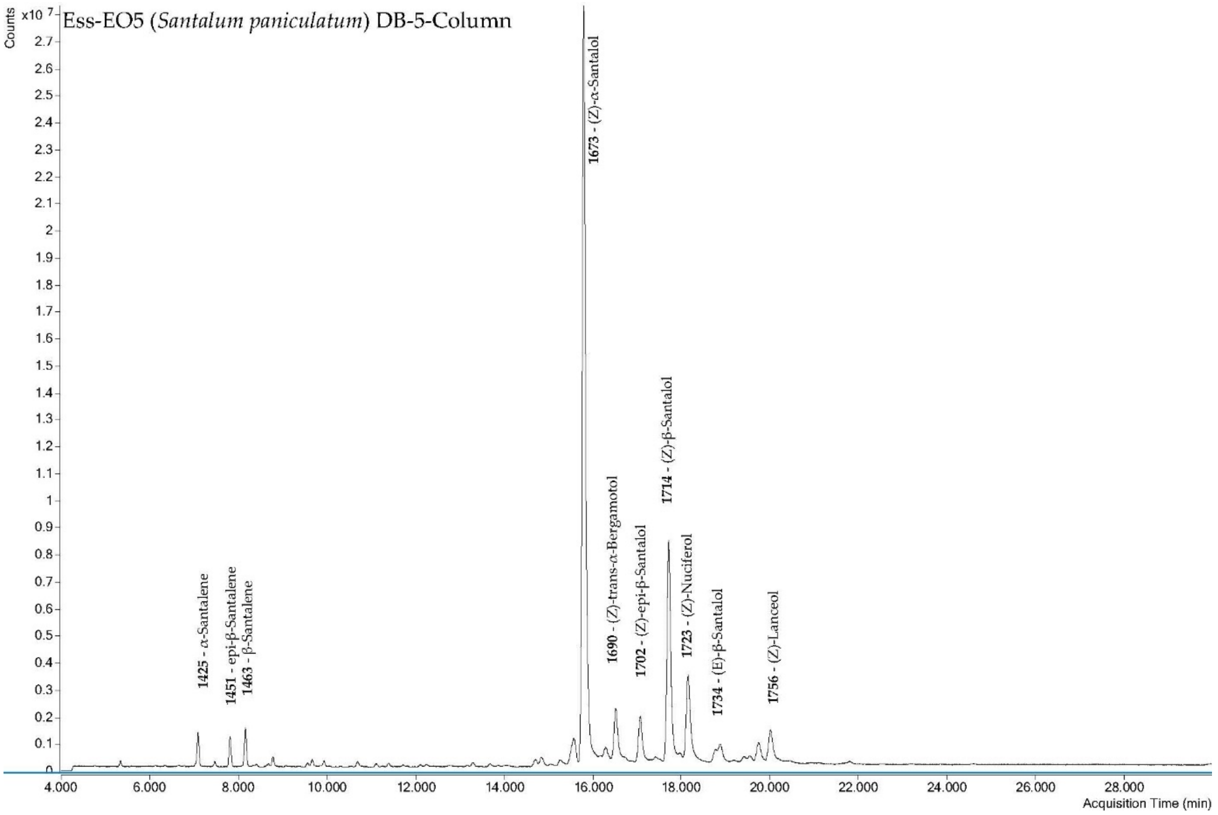

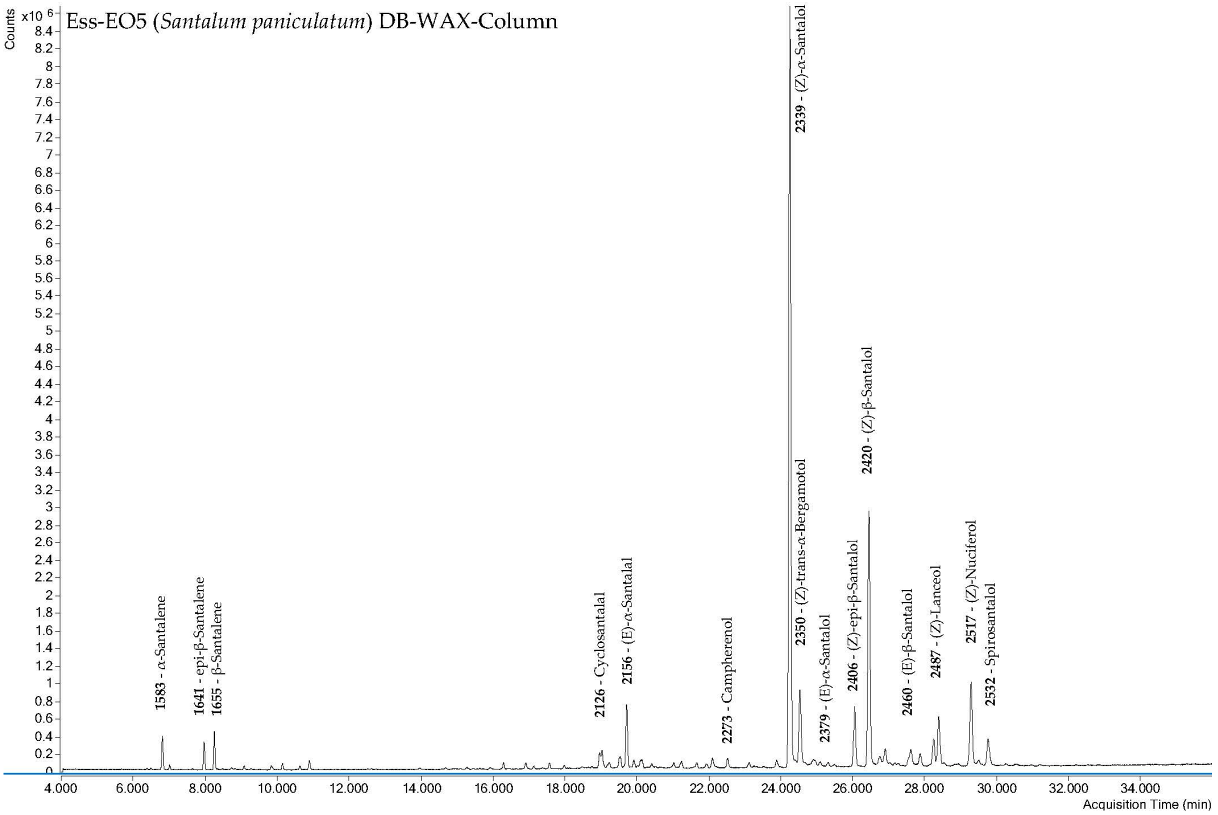

Figure S7. GC-MS chromatograms of Ess-EO5 on DB-5 and DB-HeavyWAX-columns

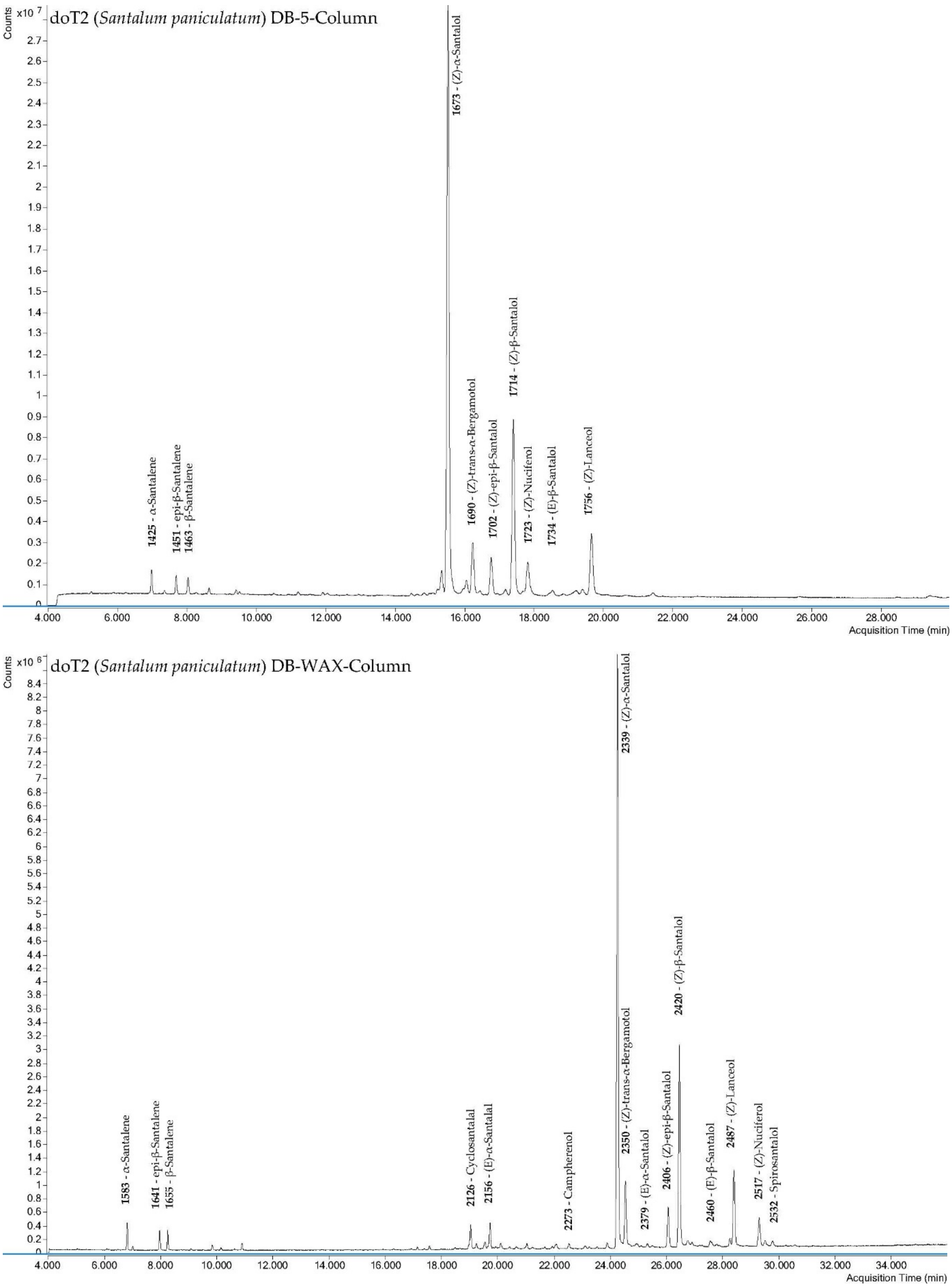

Figure S8. GC-MS chromatograms of doT2 on DB-5 and DB-HeavyWAX-columns

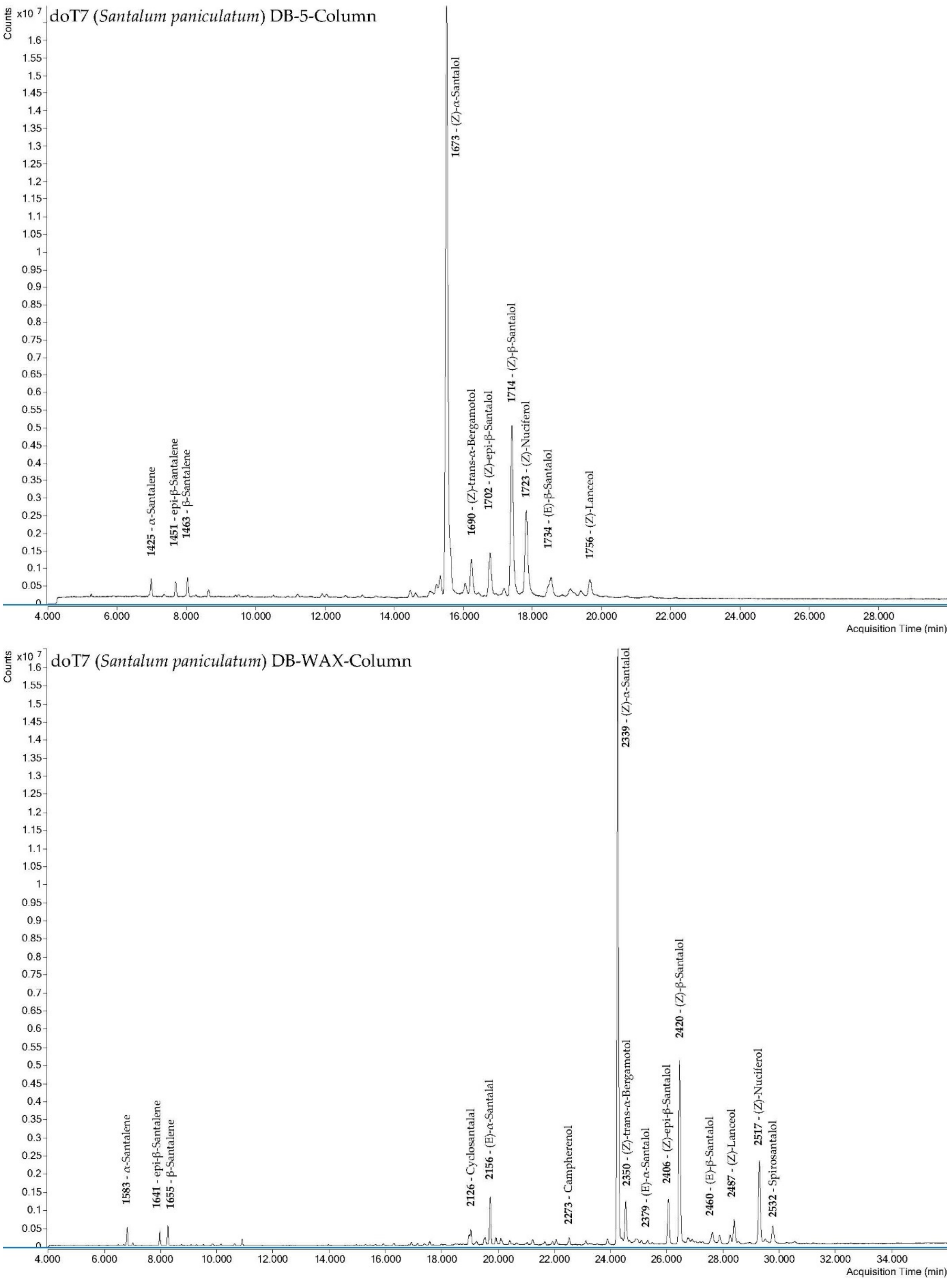

Figure S9. GC-MS chromatograms of doT7 on DB-5 and DB-HeavyWAX-columns

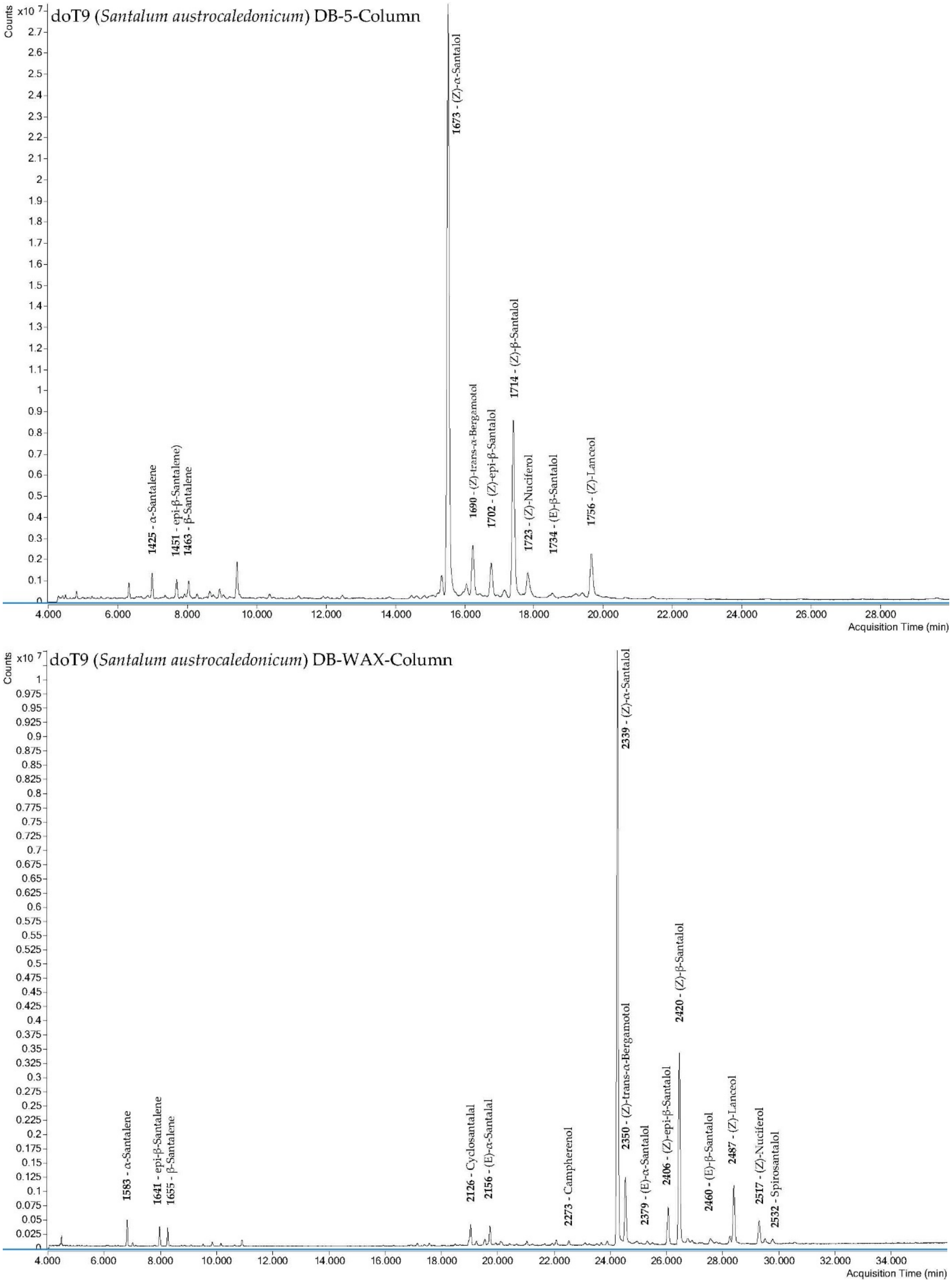

Figure S10. GC-MS chromatograms of doT9 on DB-5 and DB-HeavyWAX-columns

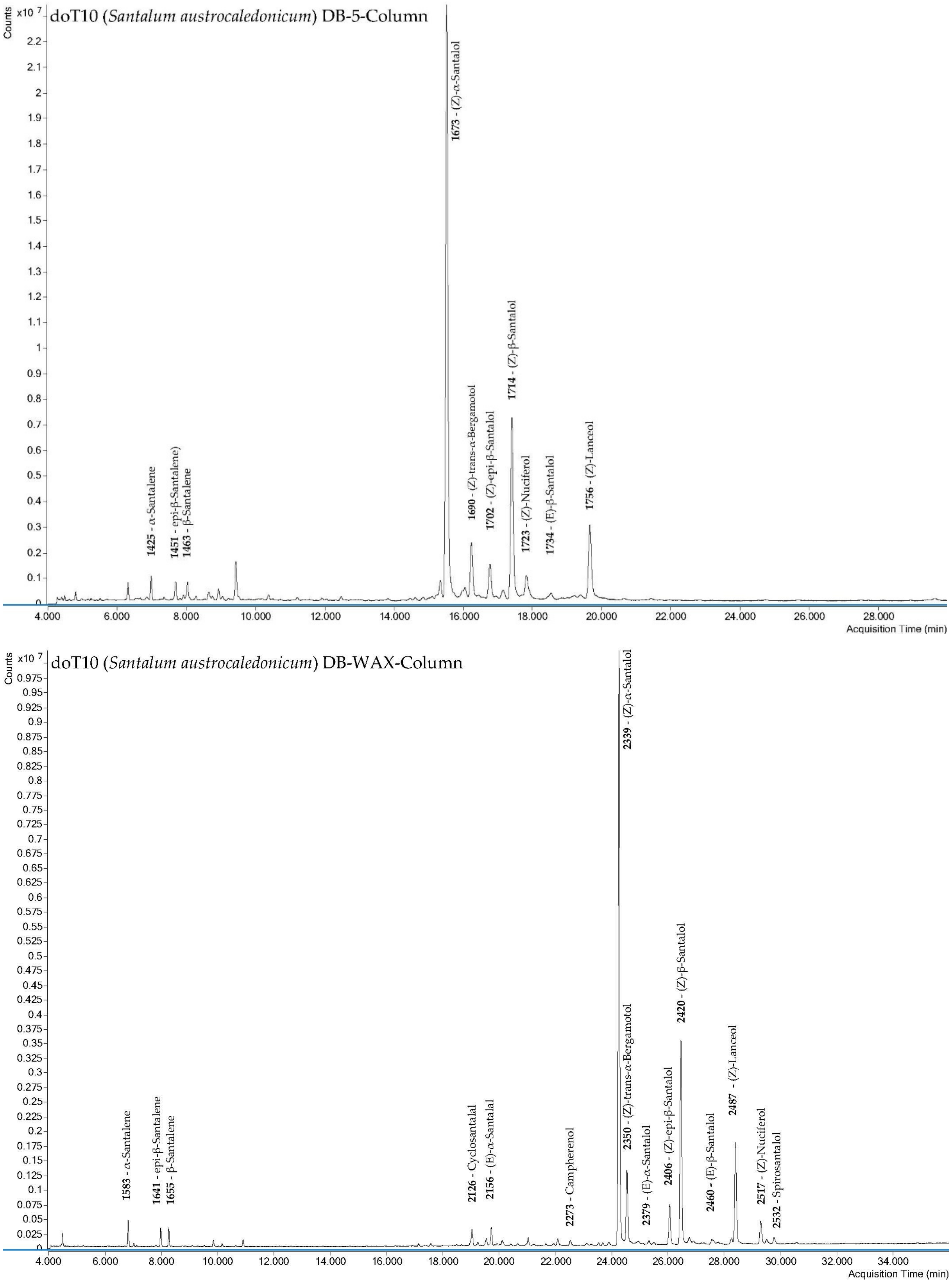

Figure S11. GC-MS chromatograms of doT10 on DB-5 and DB-HeavyWAX-columns

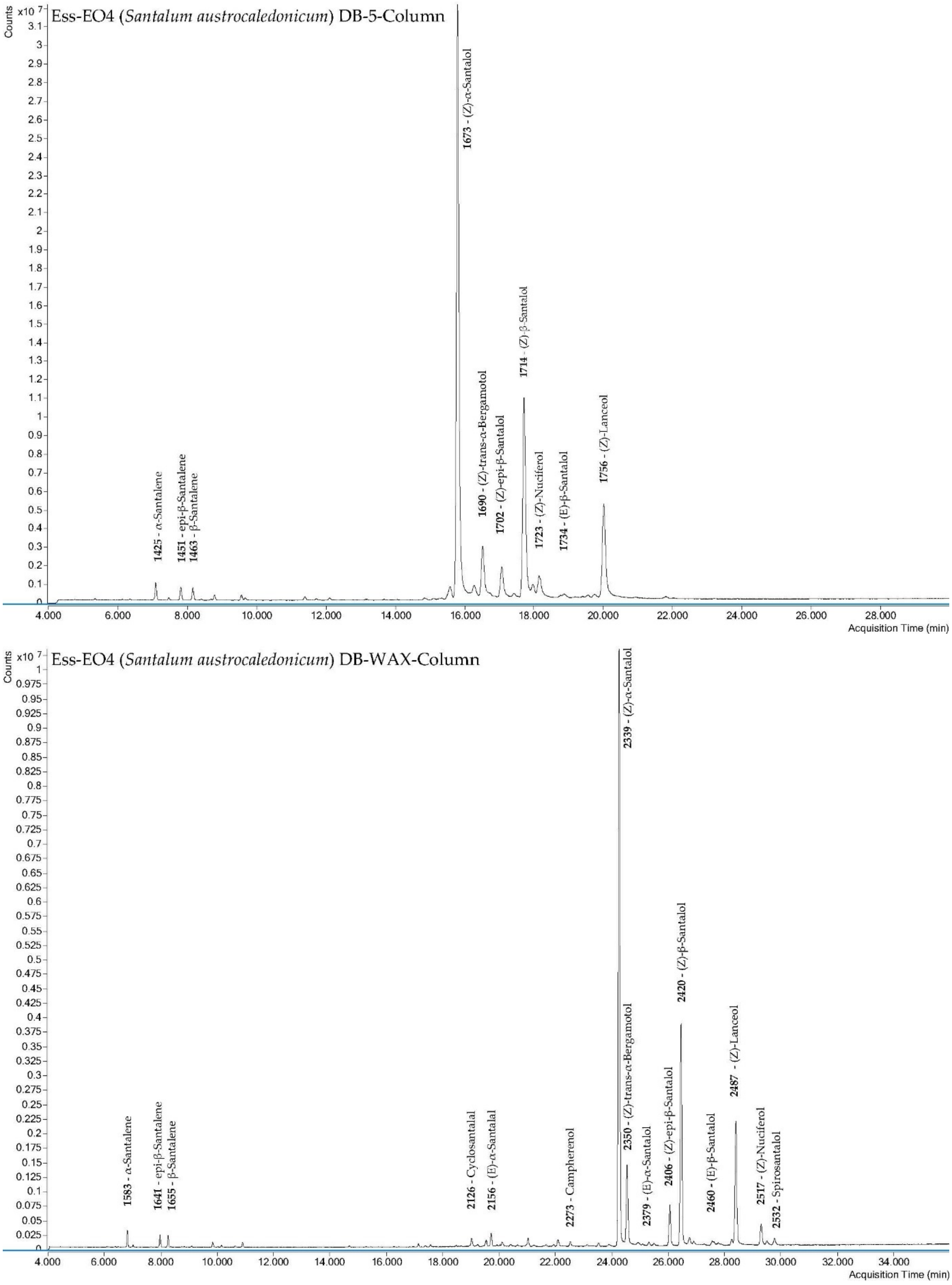

Figure S12. GC-MS chromatograms of Ess-EO4 on DB-5 and DB-HeavyWAX-columns

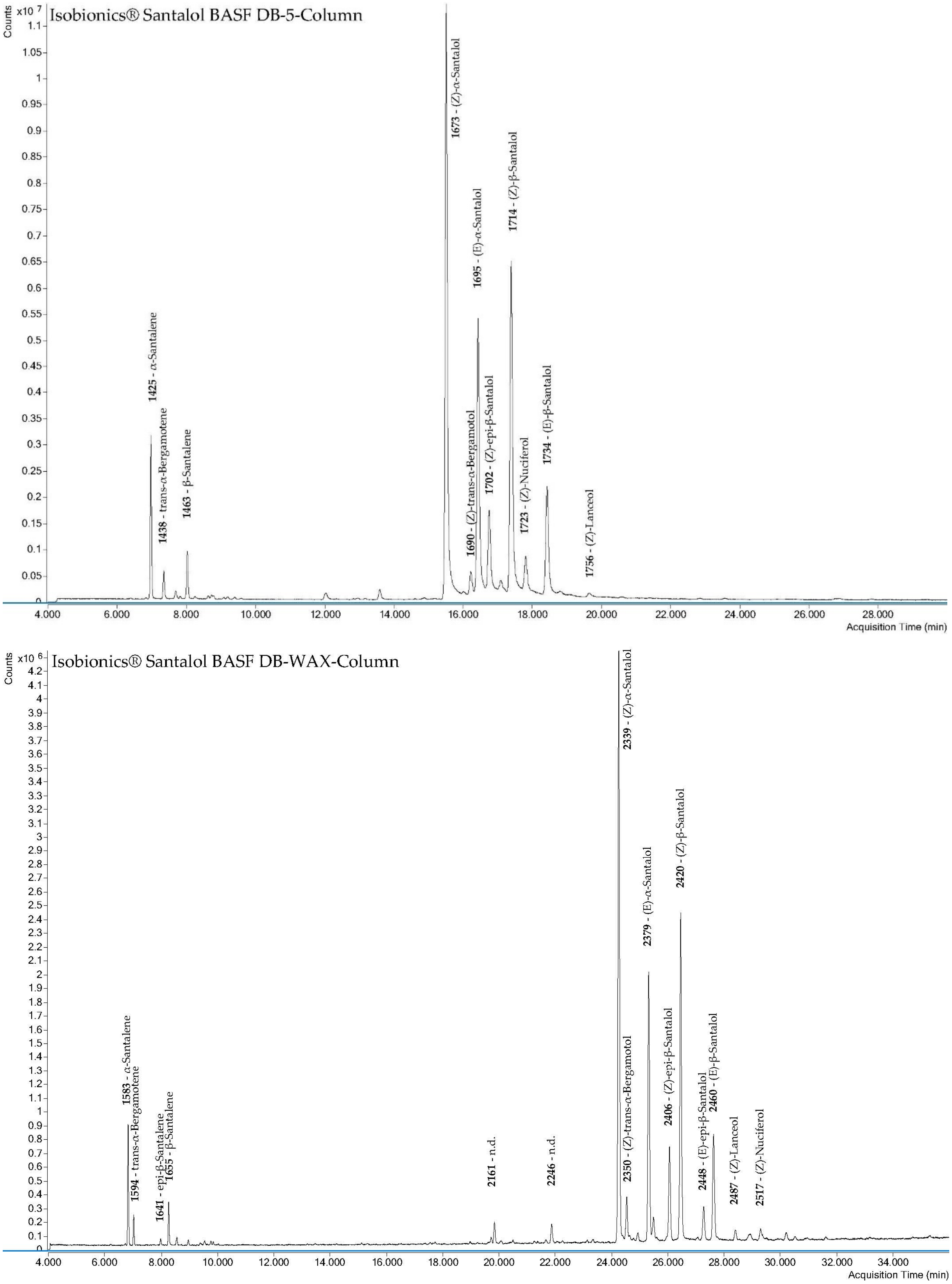

Figure S13. GC-MS Chromatograms of Isobionics® Santalol on DB-5 and DB-HeavyWAX-columns

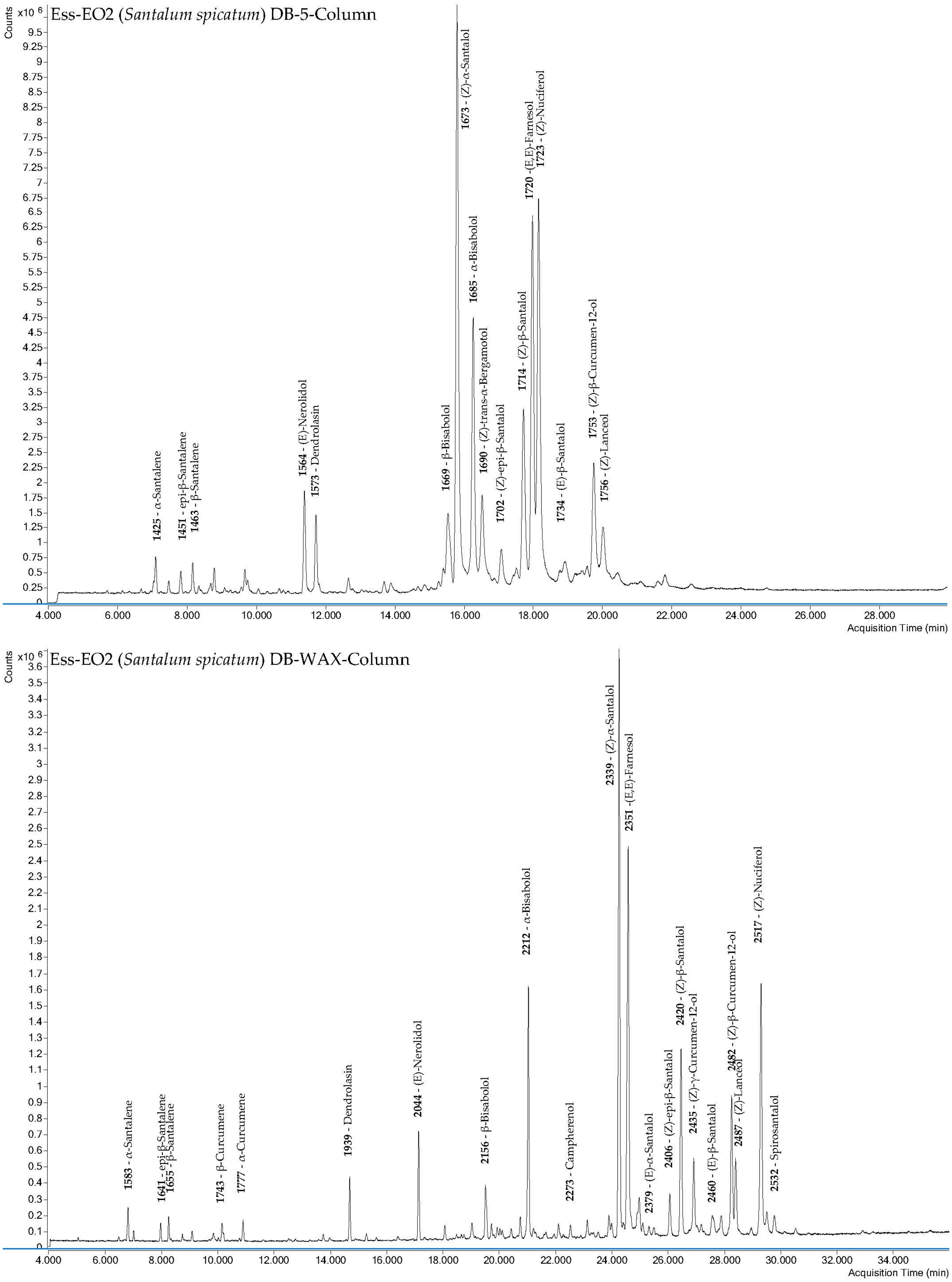

Figure S14. GC-MS chromatograms of Ess-EO2 on DB-5 and DB-HeavyWAX-columns

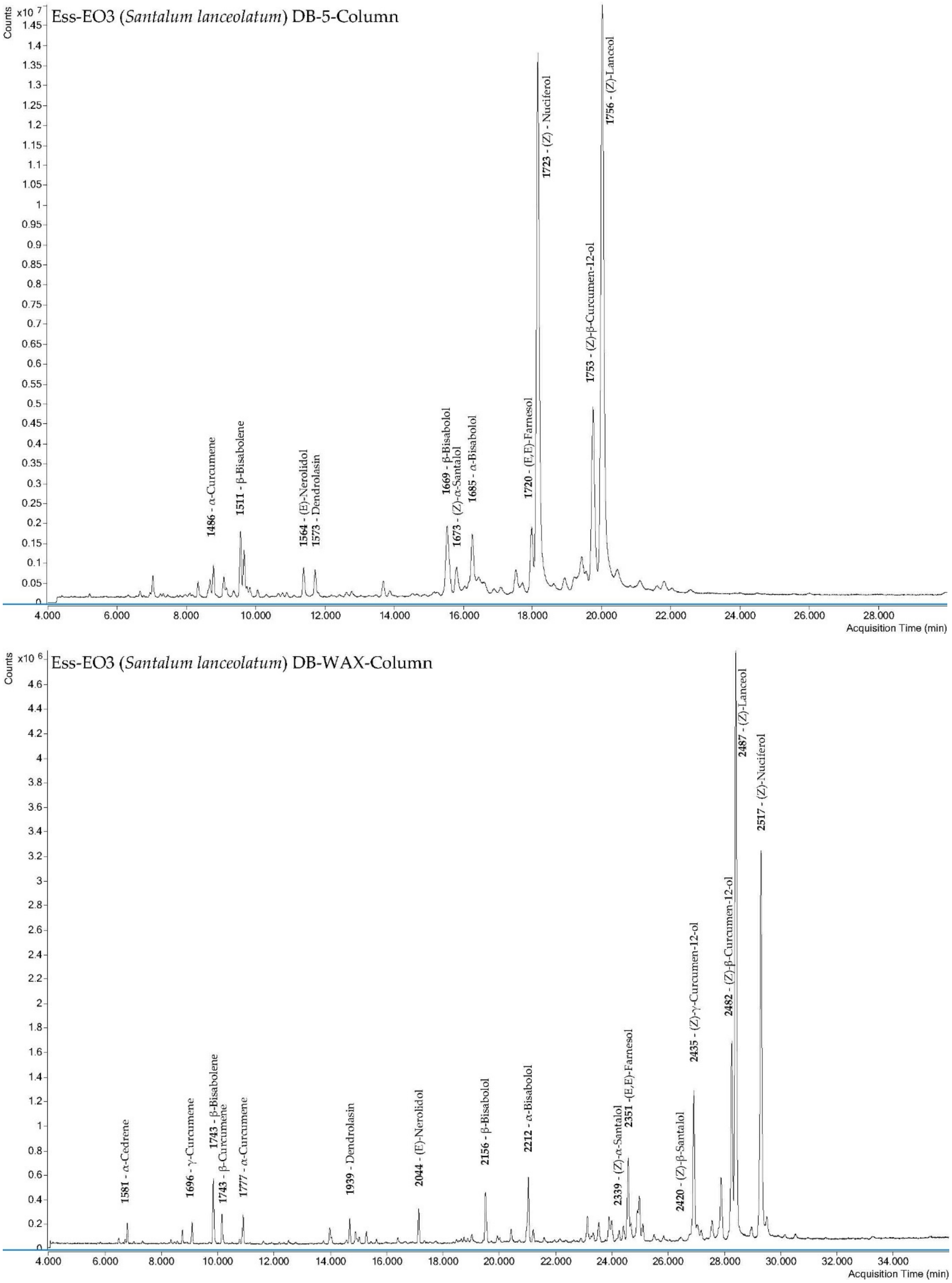

Figure S15. GC-MS chromatograms of Ess-EO3 on DB-5 and DB-HeavyWAX-columns

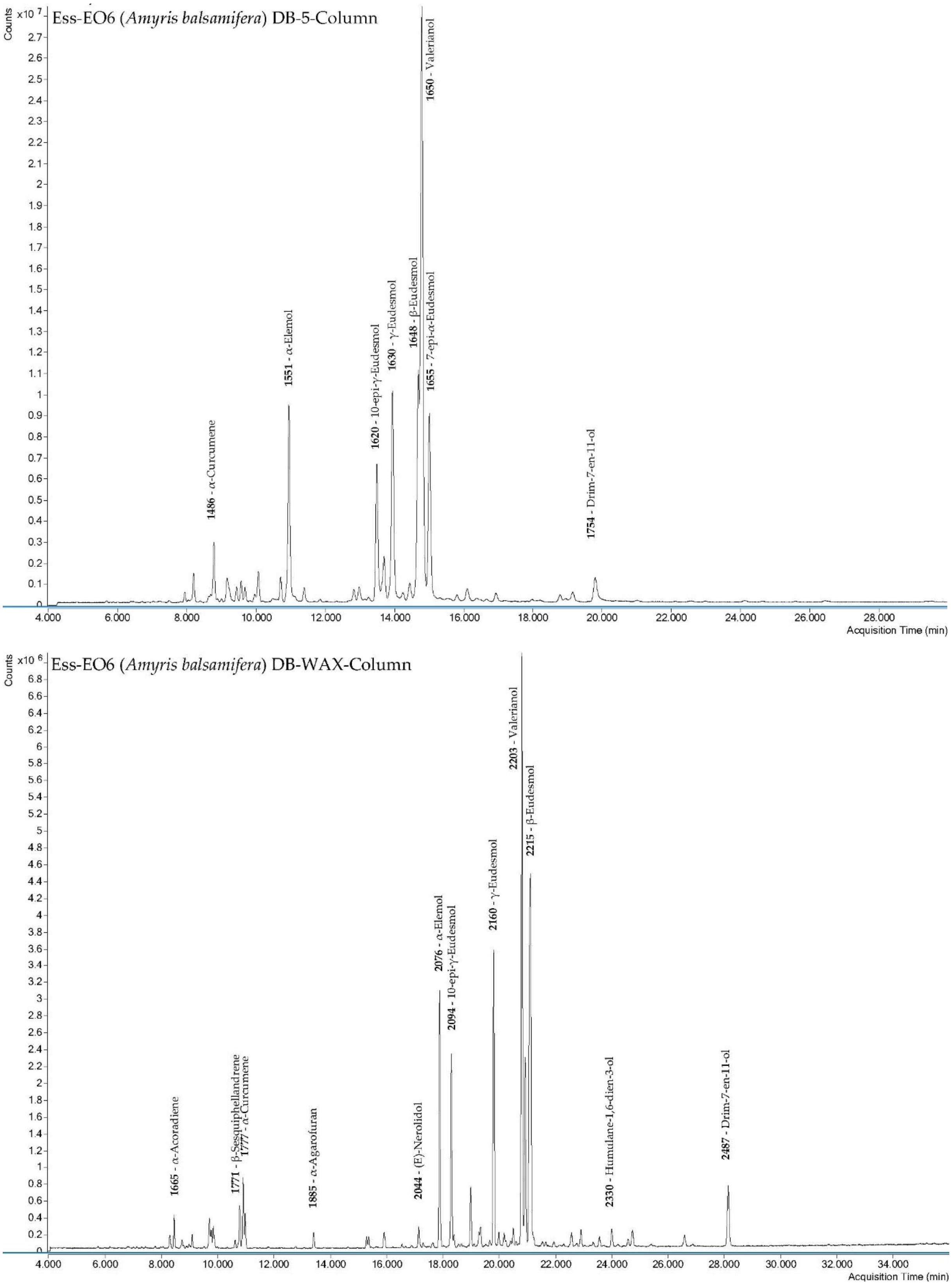

Figure S16. GC-MS chromatograms of Ess-EO6 on DB-5 and DB-HeavyWAX-columns

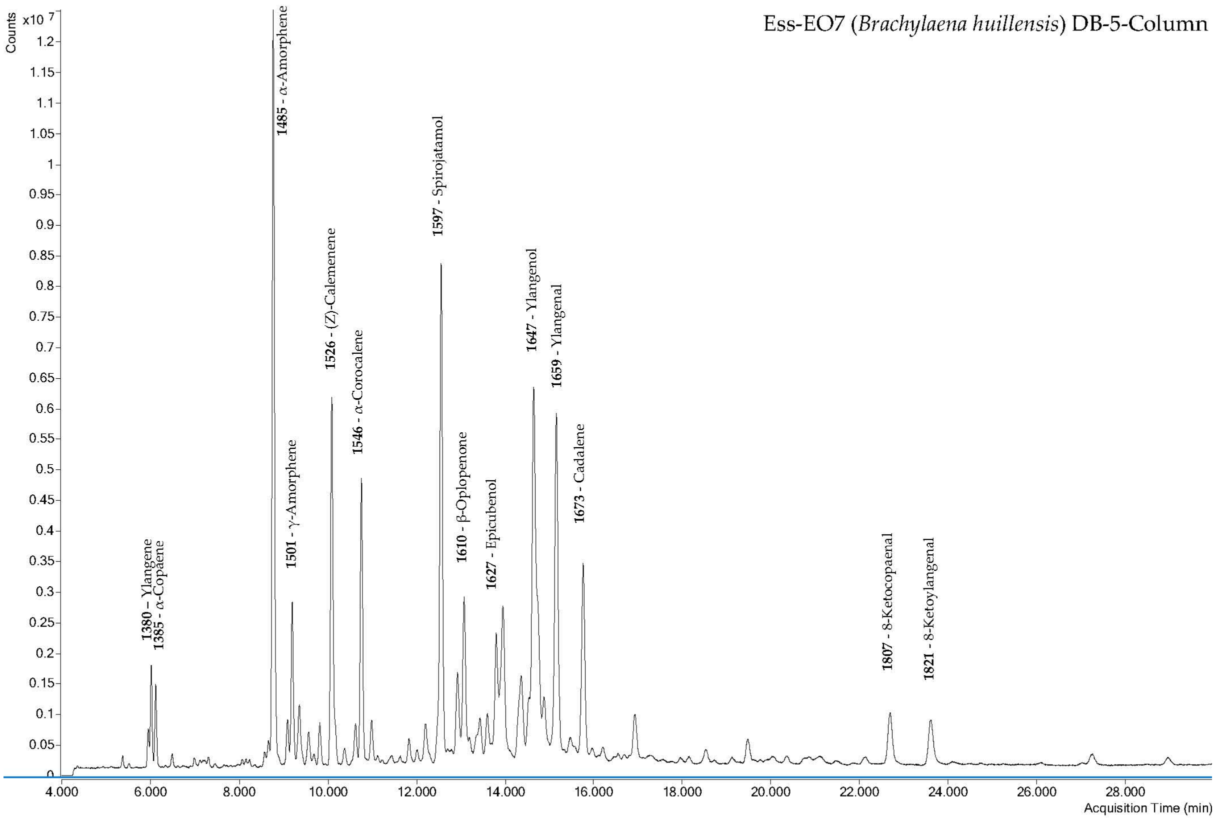

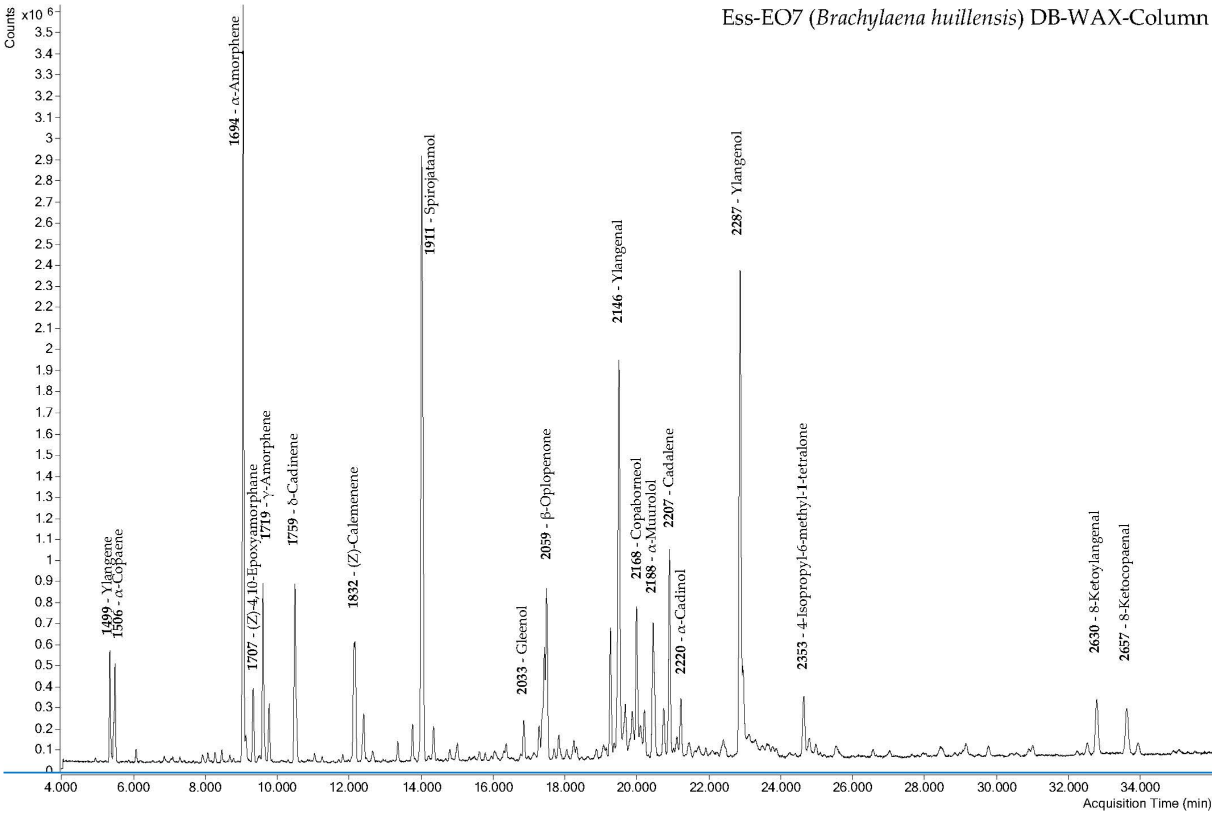

Figure S17. GC-MS chromatograms of Ess-EO7 on DB-5 and DB-HeavyWAX-columns

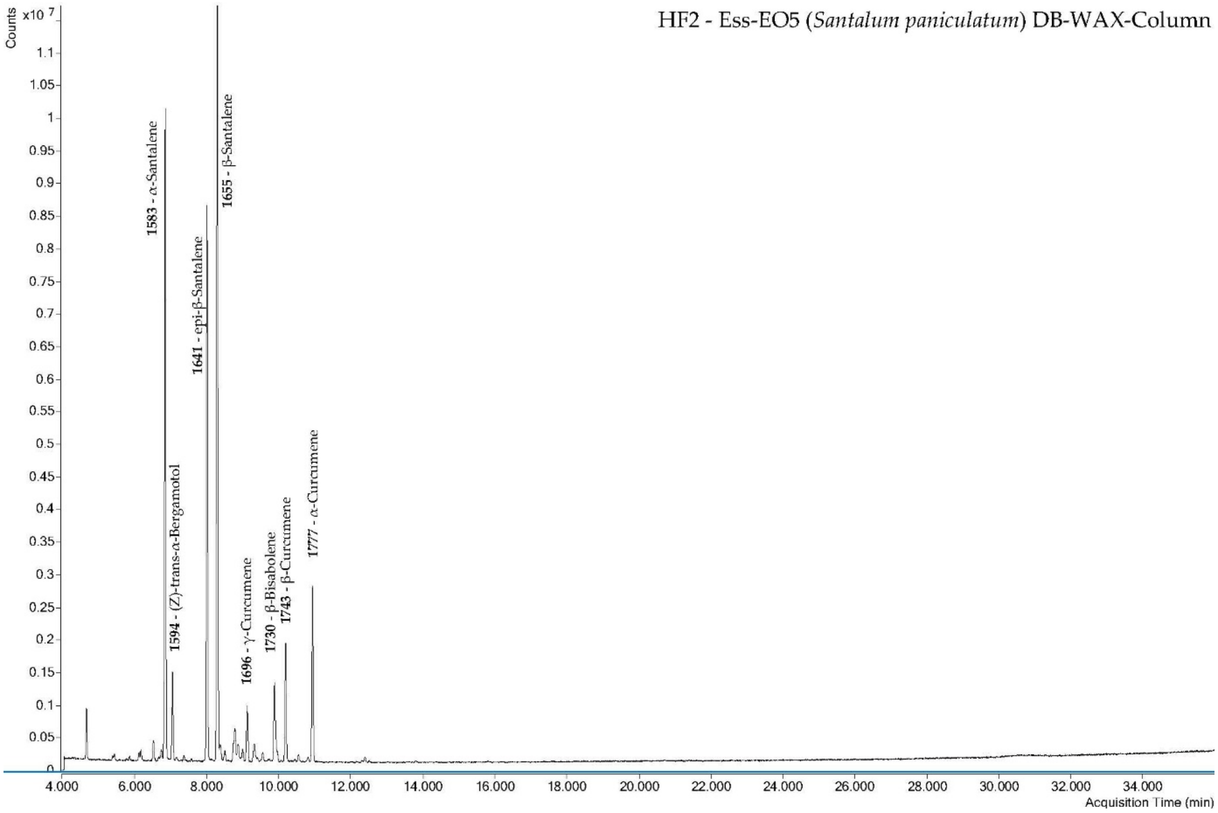

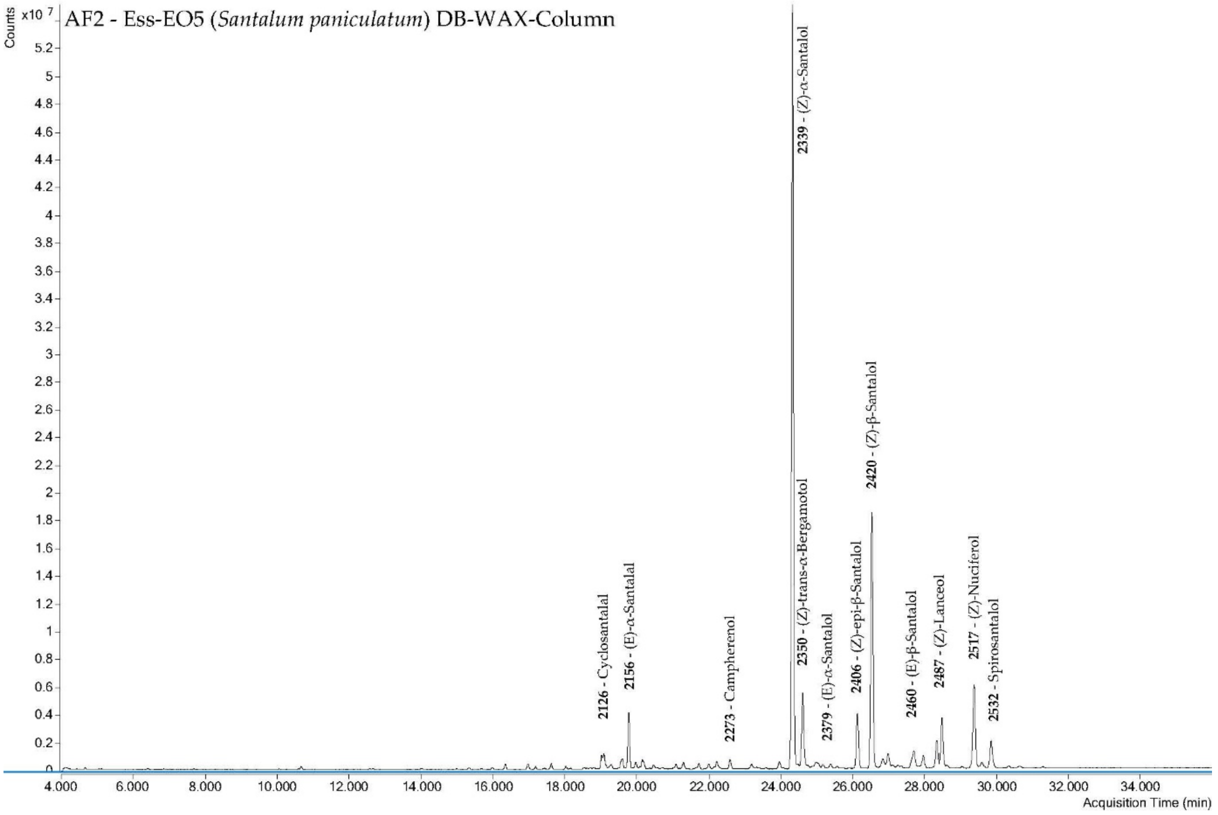

Figure S18. GC-MS chromatograms of hydrocarbon fraction (HF2) and alcohol fraction (AF2) on DB-HeavyWAX-column

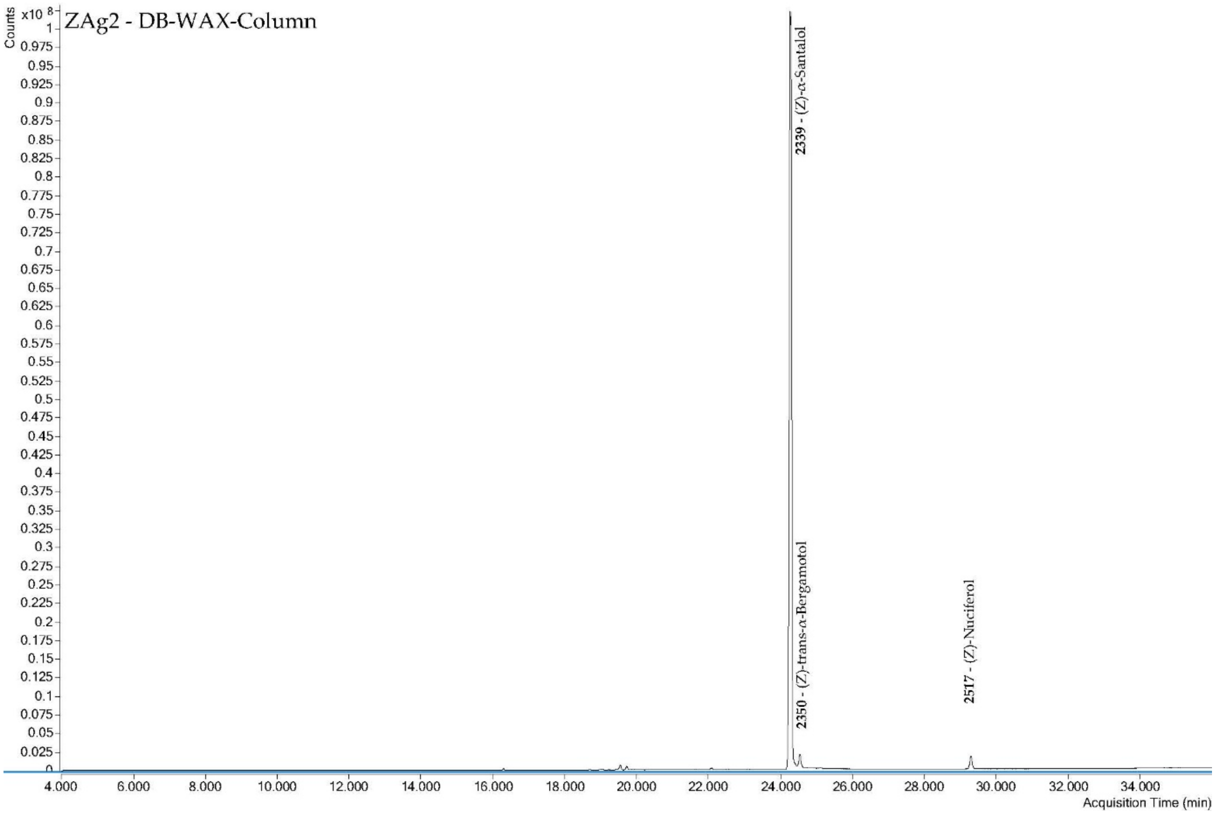

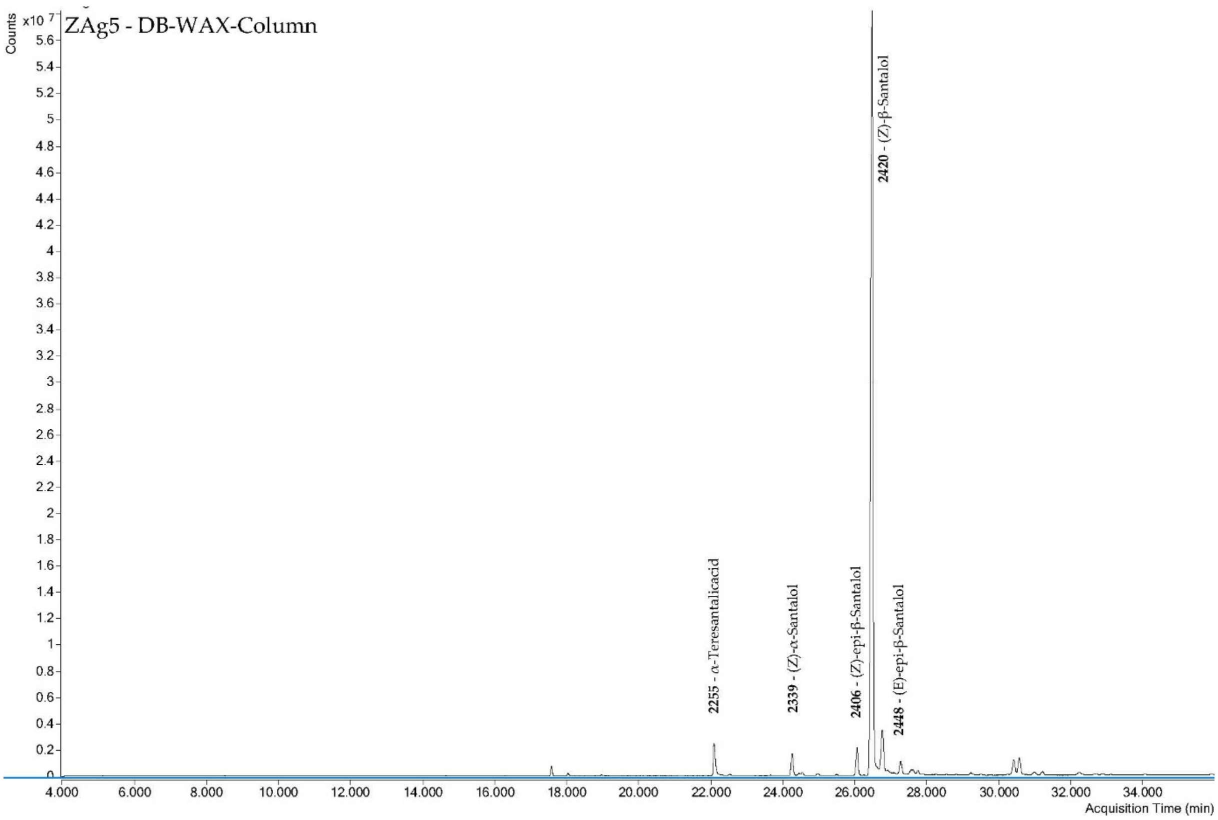

Figure S19. GC-MS chromatograms of (*Z*)-α-Santalol fraction (ZAg2, above) and (*Z*)-β-Santalol fraction (ZAg5, below) on DB-HeavyWAX-column

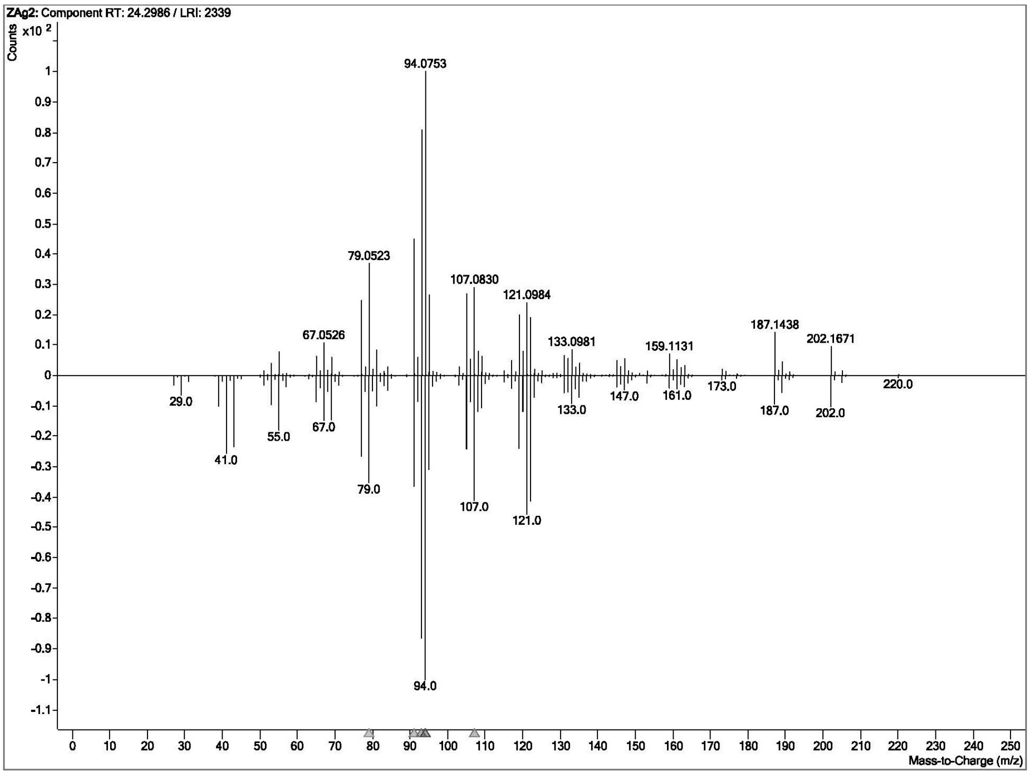

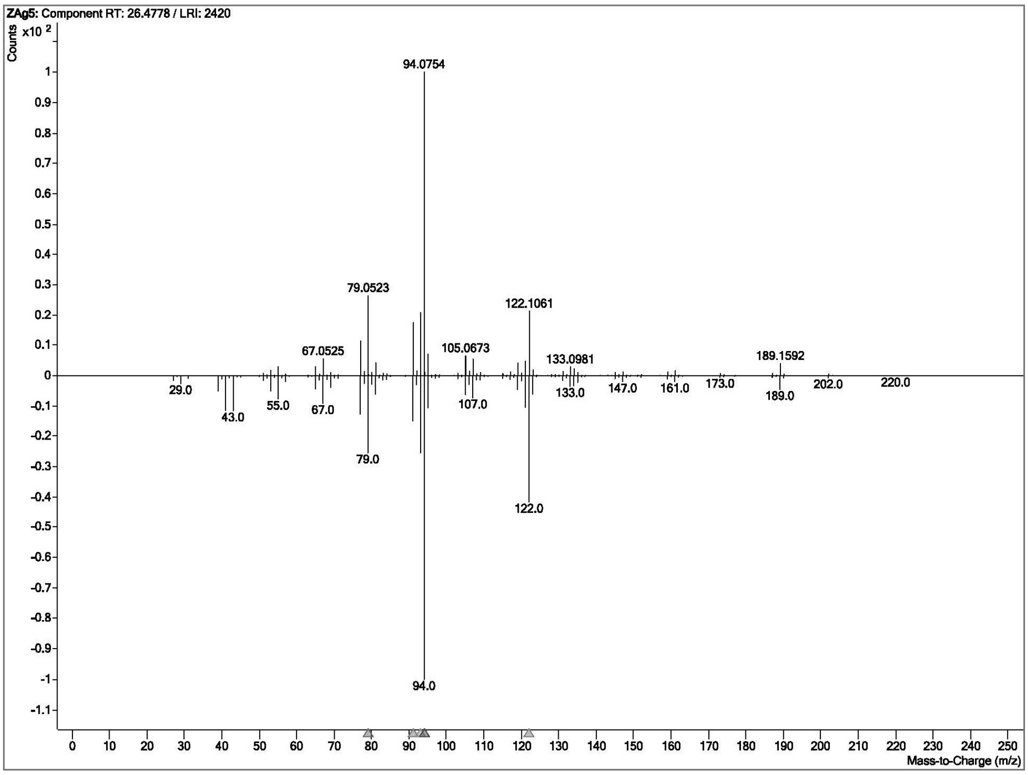

Figure S20. GC-QTOF Mass spectra of isolated (*Z*)-α-Santalol (fraction ZAg2, above) and (*Z*)-β-Santalol (fraction ZAg5, below). Measured spectra are plotted upward in direct comparison with NIST library spectra, pointing downward.
